# The oncogenic transcription factor c-Jun regulates glutaminase expression and sensitizes cells to glutaminase-targeted therapy

**DOI:** 10.1038/ncomms11321

**Published:** 2016-04-18

**Authors:** Michael J. Lukey, Kai Su Greene, Jon W. Erickson, Kristin F. Wilson, Richard A. Cerione

**Affiliations:** 1 Department of Molecular Medicine, Cornell University, Ithaca, New York 14853, USA; 2 Department of Chemistry and Chemical Biology, Cornell University, Ithaca, New York 14853, USA

## Abstract

Many transformed cells exhibit altered glucose metabolism and increased utilization of glutamine for anabolic and bioenergetic processes. These metabolic adaptations, which accompany tumorigenesis, are driven by oncogenic signals. Here we report that the transcription factor c-Jun, product of the proto-oncogene *JUN*, is a key regulator of mitochondrial glutaminase (GLS) levels. Activation of c-Jun downstream of oncogenic Rho GTPase signalling leads to elevated *GLS* gene expression and glutaminase activity. In human breast cancer cells, GLS protein levels and sensitivity to GLS inhibition correlate strongly with c-Jun levels. We show that c-Jun directly binds to the *GLS* promoter region, and is sufficient to increase gene expression. Furthermore, ectopic overexpression of c-Jun renders breast cancer cells dependent on GLS activity. These findings reveal a role for c-Jun as a driver of cancer cell metabolic reprogramming, and suggest that cancers overexpressing *JUN* may be especially sensitive to GLS-targeted therapies.

## Abstract (web summary)

Cancer cells have previously been shown to be addicted to glutamine and glutaminase enzyme activity. Here, the authors show that overexpression of the *JUN* proto-oncogene in breast cancer cells regulates *glutaminase* expression and is sufficient to confer sensitivity to glutaminase-targeted therapy.

## Graphical abstract

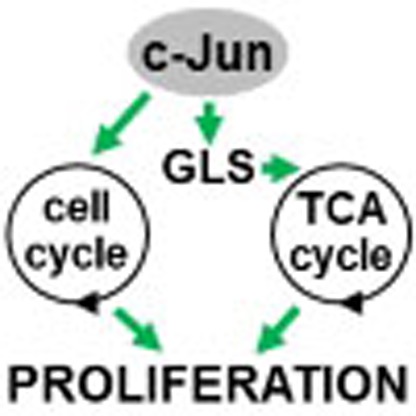

The onset of proliferation imposes a range of biosynthetic and bioenergetic demands on mammalian cells, which are met by a fundamental reprogramming of cellular metabolism^1^,^2^. The metabolic phenotype of proliferating cells, including cancer cells, typically includes high rates of glucose uptake and glycolysis coupled to lactate secretion (the Warburg effect)^3^, elevated *de novo* nucleotide biosynthesis^4^, and a high flux of mitochondrial glutamine oxidation^5^,^6^,^7^. Increased nutrient uptake and re-routing of metabolites into anabolic processes are not passive adaptations to the proliferative state, but instead are tightly regulated by the signal transduction pathways and transcriptional networks that promote cell growth and cell cycle progression. Thus, many of the oncogenic signals that lead to cellular transformation directly impact cancer cell metabolism^8^.

Metabolic reprogramming supports the proliferative state but can render cancer cells ‘addicted' to certain nutrients, and therefore provides opportunities for novel therapeutic interventions^9^. Some cancer cells show an absolute requirement for an exogenous supply of glutamine, the most abundant amino acid in plasma. Glutamine has many metabolic fates inside the cell, acting as a carbon and nitrogen source for biosynthetic reactions and also contributing to redox homoeostasis^5^,^6^,^7^. However, it is the role of glutamine as an anaplerotic substrate for the tricarboxylic acid (TCA) cycle that underlies the ‘glutamine addiction' of many rapidly proliferating cells^10^,^11^. The sequential conversion of glutamine to glutamate, and then to the TCA cycle intermediate α-ketoglutarate (α-KG), provides a mechanism for replenishing carbon that is lost from the cycle to anabolic pathways. The first reaction is catalysed by the mitochondrial enzyme glutaminase (GLS), and the second reaction by glutamate dehydrogenase or by one of several transaminase enzymes.

Two genes, *GLS* and *GLS2*, encode glutaminase enzymes in mammals^12^. The *GLS* gene encodes two splice variants, referred to as kidney-type glutaminase and glutaminase C (GAC), while the *GLS2* gene encodes two proteins through a surrogate promoter mechanism, liver-type glutaminase and GAB^12^. Whereas the GLS2 isozymes are downregulated in several cancers^13^, the GLS isozymes, in particular the GAC splice variant, are frequently upregulated in cancers of the breast^14^, lung^15^, colon^16^, prostate^17^ and brain^18^. Recently, two classes of small-molecule inhibitors of GLS have been identified, based on the lead compounds bis-2-(5-phenylacetamido-1,2,4-thiadiazol-2-yl)ethyl sulfide (BPTES) and 968 (refs 19, 20). Inhibition of GLS by these molecules, or siRNA-mediated knockdown of GLS, severely impacts the proliferation and/or survival of several cancer cell lines, but does not appear to have detrimental effects on non-tumorigenic cells^20^,^21^. Thus, there is considerable interest in targeting GLS as a therapeutic strategy against cancer, and the BPTES derivative CB-839 is currently undergoing clinical trials^21^.

One regulator of *GLS* expression and glutamine catabolism is the transcription factor c-Myc^22^,^23^. In P493 Burkitt's lymphoma and PC3 prostate cancer cell lines, c-Myc upregulates GLS through an indirect mechanism involving transcriptional repression of micro-RNAs miR-23a/b, which target the 3′-UTR of the *GLS* transcript and suppress its translation^23^. However, the relationship between c-Myc and glutamine metabolism is complex and tissue specific^24^, and tumour-specific alternative polyadenylation of the *GLS* transcript can cause a switch of the 3′-UTR, allowing it to escape c-Myc/miR-23-mediated regulation^25^. An apparent uncoupling of c-Myc and GLS has recently been described in human mammary epithelial cells as well as in certain breast cancer cell lines^26^,^27^.

We previously reported that mitochondrial glutaminase activity becomes elevated during Rho GTPase-mediated cellular transformation^20^. Here we show that the oncogenic transcription factor c-Jun is essential for this signalling outcome, and also acts as a primary regulator of *GLS* expression in human breast cancer cells. Moreover, we demonstrate that overexpression of the *JUN* proto-oncogene is sufficient to sensitize breast cancer cells to glutaminase-targeted therapy.

## Results

### Rho GTPases can drive glutamine-dependent transformation

**Figure 1:**
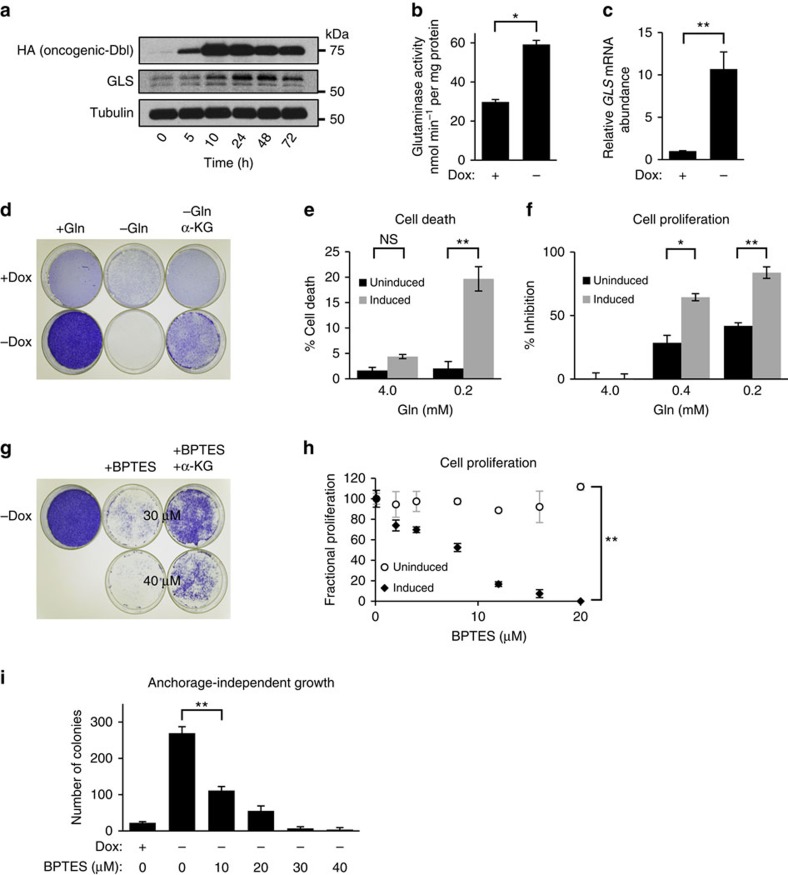
Glutamine-dependent transformation by oncogenic-Dbl. (**a**) Western blot analysis showing timecourse of oncogenic-Dbl expression in an inducible MEF system, and downstream elevation of GLS levels. Cells were induced by plating in doxycycline-free growth medium, and samples were collected at time-points up to 72 h. (**b**) Glutaminase activity assay using mitochondria isolated from MEFs in which oncogenic-Dbl expression was either uninduced (+Dox) or induced for 24 h (−Dox). Activity is expressed per mg of total cellular protein, and data presented are the mean±s.d. of triplicate assays. (**c**) RT–PCR analysis of uninduced and induced (24 h) MEFs, showing relative levels of the *GLS* transcript. The data presented are the RQ values, with error bars marking RQ max and RQ min, from triplicate reactions. (**d**) Saturation density analysis showing the effect of glutamine withdrawal on oncogenic-Dbl inducible MEFs. Dishes of uninduced (+Dox) and induced (−Dox) cells cultured in 4 mM glutamine, or in glutamine-free medium ±2 mM dimethyl α-ketoglutarate, were fixed and then stained with crystal violet. (**e**) Cell death analysis for uninduced or induced cells after 6 days culture in 4.0 or 0.2 mM glutamine. Data presented are the mean±s.d. of triplicate assays. (**f**) Cell proliferation assays showing the effect of glutamine depletion on proliferation of uninduced and induced MEFs over 6 days. Data presented are the mean±s.d. of triplicate assays. (**g**) Saturation density analysis showing the effect of the GLS inhibitor BPTES on oncogenic-Dbl induced MEFs. Induced (−Dox) cells cultured in the absence or presence of BPTES (30 or 40 μM)±2 mM dimethyl α-ketoglutarate were fixed and then stained with crystal violet. (**h**) BPTES dose curves showing the effect of different BPTES concentrations on proliferation over 6 days of uninduced or induced MEFs. Fractional proliferation relative to untreated cells is shown. Assays were carried out in 10% FBS medium, and data presented are the mean±s.d. of triplicate assays. (**i**) Anchorage-independent growth assay for uninduced (+Dox) cells, and for induced (−Dox) cells cultured under increasing BPTES concentrations. Data presented are the mean±s.d. of triplicate assays. Differences were analysed with Student's *t*-test. *P<0.05, **P<0.01.

We previously reported that oncogenic-Dbl, a guanine nucleotide exchange factor and potent activator of Rho GTPases, signals to upregulate mitochondrial GLS activity in NIH/3T3 cells^20^. This is an essential event for maintaining Dbl-induced cellular transformation. To explore further the signalling connections that link Rho GTPases with GLS, we utilized an inducible, tetracycline-off, system to control the expression of oncogenic-Dbl in mouse embryonic fibroblasts (MEFs). When doxycycline (0.6 μg ml^−1^) is present in the culture medium, HA-tagged oncogenic-Dbl is undetectable by western blot analysis of whole-cell lysates. Removal of doxycycline triggers a robust expression of oncogenic-Dbl within 10 h that remains elevated through 72 h (Fig. 1a). This is accompanied by a corresponding increase in GLS protein levels, which peak 24–48 h following induction (Fig. 1a). We isolated mitochondria from uninduced and induced MEFs and assayed the preparations for glutaminase activity as described previously^28^. This confirmed that induction of oncogenic-Dbl results in elevated glutaminase activity (Fig. 1b). We then tested whether GLS was upregulated at the transcriptional level. Cells that were either uninduced or induced (24 h) were analysed by real-time PCR (RT–PCR), which revealed that induction of oncogenic-Dbl expression leads to an ∼12-fold increase of the *GLS* transcript (Fig. 1c).

Induction of oncogenic-Dbl caused MEFs to acquire transformed characteristics, including greatly increased saturation density, and the ability to proliferate in low serum (0.5% fetal bovine serum (FBS)) medium and to undergo anchorage-independent growth. Since these changes were accompanied by an increase in GLS levels, we tested whether cellular transformation was dependent on an exogenous supply of glutamine, and/or on GLS enzymatic activity. After 8 days of culture in medium supplemented with 10% FBS±4 mM glutamine, saturation density was assessed by fixing and staining cells with crystal violet (Fig. 1d). Prior to fixation, cells were imaged at × 100 magnification (Supplementary Fig. 1a). The increased saturation density of cells expressing oncogenic-Dbl was completely abolished in glutamine-free medium, but could be partially rescued by supplementation with a cell-permeable analogue of α-ketoglutarate (dimethyl-α-KG), a downstream metabolite of the GLS reaction. We compared cell viability under high-glutamine (4 mM) and low-glutamine (0.2 mM) conditions, and found that uninduced cells remained fully viable following glutamine depletion, whereas induced cells showed an ∼5-fold increase in cell death (Fig. 1e). The proliferation rate of both uninduced and induced cells decreased on glutamine deprivation, although the inhibition was ∼2-fold greater in induced cells (Fig. 1f).

We then used BPTES^19^, a small-molecule inhibitor of GLS, to test whether MEFs transformed by oncogenic-Dbl were dependent on GLS activity. Saturation density of induced cells was decreased by BPTES treatment in a dose-dependent manner, and partially rescued by supplementation with dimethyl-α-KG (Fig. 1g and Supplementary Fig. 1b). Cell proliferation assays illustrated the remarkable difference in BPTES sensitivity between uninduced and induced MEFs (Fig. 1h). Proliferation of induced cells was potently inhibited by BPTES with an IC_50_ of 8 μM, whereas uninduced cells were unaffected even by 20 μM BPTES. Oncogenic-Dbl-induced cells, but not uninduced cells, were capable of anchorage-independent growth. This too was completely blocked by BPTES treatment (Fig. 1i).

### Rho GTPases signal to c-Jun to upregulate *GLS* expression

**Figure 2:**
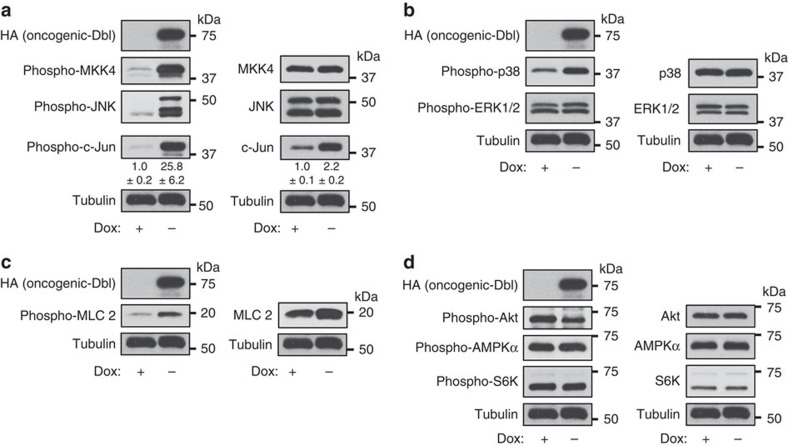
Oncogenic-Dbl potently activates the MKK4-JNK-c-Jun signalling axis in MEFs. Western blot analysis of whole-cell lysates of MEFs in which oncogenic-Dbl expression was either uninduced (+Dox) or induced for 24 h (−Dox) under low-serum (0.5% FBS) conditions. The extent of activation of different signalling pathways was assessed using phospho-specific antibodies that recognize activated kinases or their downstream phosphorylation targets (left panels). Any changes in total protein levels were also assessed (right panels). (**a**) The MAPK signalling axis involving MKK4-JNK-c-Jun is potently activated downstream of oncogenic-Dbl. Total c-Jun levels are also elevated, consistent with previous reports showing auto-regulation of *JUN* expression. (**b**) The MAPK p38 is moderately activated, whereas ERK is only slightly activated on oncogenic-Dbl induction. (**c**) ROCK activity, as read-out by MLC 2 phosphorylation, is activated on induction. (**d**) Akt is slightly inhibited, and AMPK and mTORC1 activity (the latter read-out by S6K phosphorylation) are largely unaffected by oncogenic-Dbl induction. Relative densitometry data are the mean±s.d. of triplicate blots.

The results above show that oncogenic-Dbl signals to upregulate GLS at the transcript and protein level, and that cellular transformation driven by oncogenic-Dbl is dependent on GLS activity and an exogenous supply of glutamine. We next wanted to identify the signalling pathway downstream of the Rho GTPases that was responsible for regulating *GLS* expression. We first used phospho-specific antibodies to assess the effects of oncogenic-Dbl induction on key cellular signalling proteins. Here, and throughout the study, low-serum conditions (0.5% FBS) were used for signalling experiments to minimize ‘background' cellular signalling activity. Western blot analysis of whole-cell lysates revealed an extremely potent activation of the MKK4/c-Jun N-terminal kinase (JNK) signalling axis on induction (24 h), and downstream activating phosphorylation of the oncogenic transcription factor c-Jun (Fig. 2a). c-Jun is known to autoregulate its own gene expression in MEFs^29^,^30^ and, consistent with this, we found that total c-Jun levels increased as well as phosphorylated c-Jun levels (2-fold and 26-fold, respectively) (Fig. 2a and Supplementary Fig. 2a). The JNK kinase MKK7 and the Jun-family members JunB and JunD were expressed at low levels, and phosphorylated forms could not be detected (Supplementary Fig. 2b,c). Another target of MKK4, p38, was phosphorylated downstream of oncogenic-Dbl, whereas the activation status of ERK showed only a subtle change (Fig. 2b). Rho-associated coiled-coil containing protein kinase (ROCK) was likely activated, as indicated by increased phosphorylation of its substrate myosin light chain 2 (MLC 2) (Fig. 2c). Consistent with earlier reports that ROCK signals to inhibit Akt^31^, a modest decrease in Akt phosphorylation at residue T308 occurred on induction (Fig. 2d), and this could be rescued by inhibition of ROCK (Supplementary Fig. 2d). The mTORC1-S6K signalling axis showed little change on oncogenic-Dbl induction, and we also detected no change in the phosphorylation level of AMPKα (Fig. 2d).

**Figure 3:**
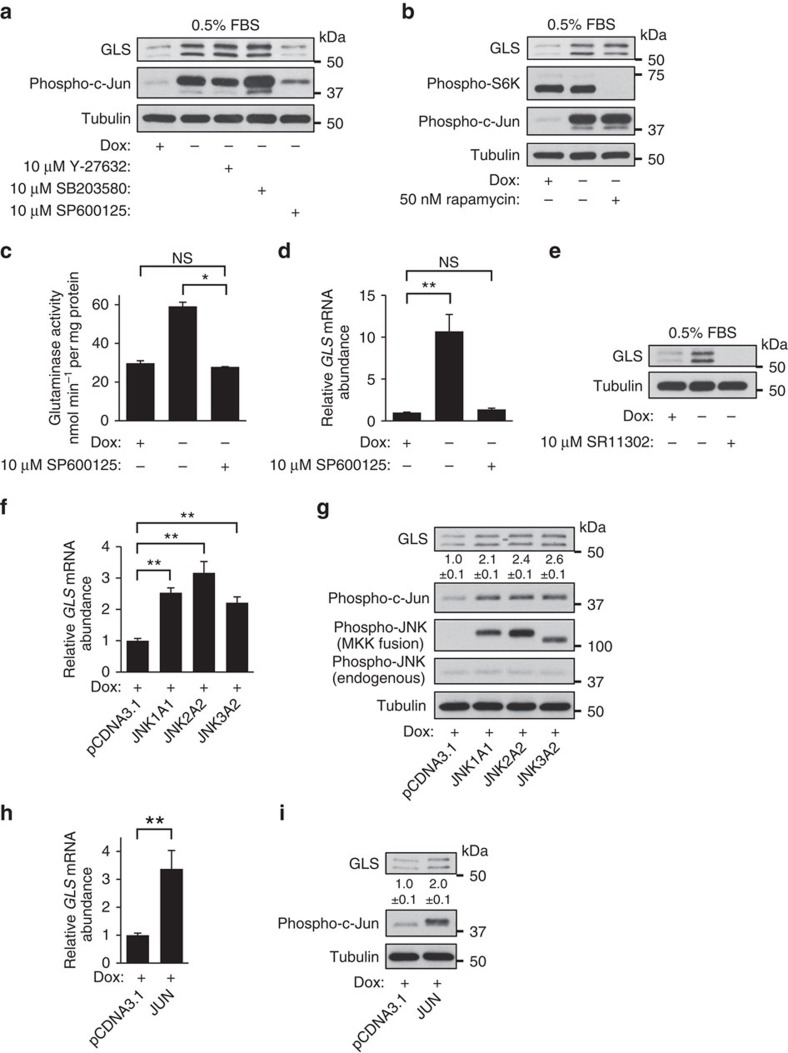
Oncogenic-Dbl signals to c-Jun to upregulate GLS in MEFs. (**a**) Western blot analysis of whole-cell lysates of uninduced (+Dox) or induced for 48 h (−Dox) MEFs±10 μM selective inhibitors of ROCK (Y-27632), p38 (SB203580) or JNK (SP600125). Inhibition of JNK largely blocks the upregulation of GLS downstream of oncogenic-Dbl. (**b**) Western blot analysis showing that inhibition of mTORC1 by rapamycin has little effect on the upregulation of GLS downstream of oncogenic-Dbl. (**c**) Glutaminase activity assay using mitochondria isolated from uninduced (+Dox) MEFs and from MEFs that had been induced for 24 h (−Dox) in the absence or presence of 10 μM SP600125 (JNK inhibitor). Activity is expressed per mg of total cellular protein and data presented are the mean±s.d. of triplicate assays. (**d**) RT–PCR analysis of uninduced (+Dox) and 24 h induced (−Dox) MEFs±10 μM SP600125, showing relative levels of the *GLS* transcript. The data presented are the RQ values, with error bars marking RQ max and RQ min, from triplicate reactions. (**e**) Western blot analysis showing that the AP-1 inhibitor SR11302 (10 μM) completely blocks *GLS* expression in oncogenic-Dbl-induced MEFs. (**f**) RT–PCR analysis showing that transient transfection of uninduced (+Dox) MEFs with constitutively activated JNK/MKK fusion constructs leads to increased *GLS* mRNA abundance. The data presented are the RQ values, with error bars marking RQ max and RQ min, from triplicate reactions. (**g**) Western blot analysis of the samples from the previous panel, showing that ectopic expression of constitutively activated JNK fusion constructs in uninduced MEFs leads to increased phosphorylation of c-Jun and upregulated GLS protein levels. (**h**) RT–PCR analysis showing that transient transfection of uninduced (+Dox) MEFs with a construct for expressing the *JUN* proto-oncogene leads to increased *GLS* mRNA abundance. The data presented are the RQ values, with error bars marking RQ max and RQ min, from triplicate reactions. (**i**) Western blot analysis of the samples from the previous panel, showing that ectopic expression of *JUN* in uninduced MEFs leads to increased levels of phospho-c-Jun and upregulated GLS protein levels. Note that the ectopically expressed c-Jun contains a V5-tag, and consequently runs at a slightly higher molecular weight than endogenous c-Jun. Relative densitometry data are the mean±s.d. of triplicate blots. Differences were analysed with Student's *t*-test. *P<0.05, **P<0.01.

Since ROCK, p38 and JNK signalling were all activated downstream of oncogenic-Dbl, we tested whether selective inhibition of these kinases (by the small-molecule inhibitors Y-27632, SB203580 and SP600125, respectively) impacted the ability of oncogenic-Dbl to upregulate GLS. Cells were induced for 48 h, either in the absence or presence of 10 μM of each inhibitor, and whole-cell lysates were then analysed by western blot. Treatment of induced cells with the ROCK inhibitor Y-27632 or the p38 inhibitor SB203580 did not significantly impact GLS levels, whereas the JNK inhibitor SP600125 largely blocked the upregulation of GLS (Fig. 3a). Although rapamycin-sensitive mTORC1 can influence GLS levels by increasing *MYC* translation^32^, we found that regulation of GLS downstream of the Rho GTPases was independent of mTORC1 activity (Fig. 3b), and changes in c-Myc (and phospho-c-Myc) levels did not fully correspond to changes in GLS (Supplementary Fig. 2e). Treatment of induced cells with the c-Myc inhibitor 10058-F4 severely impacted viability in low-serum (0.5% FBS) culture medium (no viable cells remained after 24 h treatment with 10 μM 10058-F4), and it is possible that c-Myc contributes to the regulation of *GLS* expression by Rho GTPases under these conditions. In high-serum (10% FBS) medium, treatment with 10058-F4 at concentrations up to 60 μM had little impact on GLS levels in induced cells (Supplementary Fig. 2f), whereas inhibition of JNK by 10 μM SP600125 still suppressed *GLS* expression (Supplementary Fig. 2e).

Consistent with the effects on GLS protein levels, treatment with the JNK inhibitor SP600125 (10 μM) completely blocked the increase in mitochondrial glutaminase activity that occurs downstream of oncogenic-Dbl signalling (Fig. 3c). Because JNK directly phosphorylates activator protein 1 (AP-1) family transcription factors including the archetypal substrate c-Jun, leading to increased transcriptional activity, we tested whether blockade of JNK signalling affected *GLS* transcript levels. The ∼12-fold increase in *GLS* mRNA that occurs following oncogenic-Dbl induction was completely abolished by 10 μM SP600125 (Fig. 3d). To test the hypothesis that the effects of JNK inhibition were mediated by suppression of c-Jun transcriptional activity, we treated induced cells with 10 μM SR11302, a selective inhibitor of the AP-1 family of transcription factors to which c-Jun belongs^33^. This resulted in the complete loss of GLS to undetectable levels (Fig. 3e).

The experiments above suggested that the signal to elevate *GLS* expression downstream of oncogenic-Dbl is transmitted via JNK-mediated phosphorylation of a Jun-family AP-1 transcription factor, most likely c-Jun, since it is highly expressed in these cells (Fig. 2a), in contrast to JunB and JunD (Supplementary Fig. 2c). To test whether JNK and c-Jun can upregulate *GLS* expression, we transiently transfected uninduced MEFs with constructs for expressing constitutively activated MKK/JNK fusion proteins^34^ or c-Jun. Uninduced cells were cultured for 48 h after transfection (in 10% FBS medium, as transfection reagents harmed viability of MEFs in 0.5% FBS medium), and then analysed by RT–PCR and western blot. Ectopic expression of JNK1, JNK2 or JNK3 fusion-constructs all led to elevated *GLS* transcript levels relative to vector-control cells (Fig. 3f). Western blot analyses of whole-cell lysates showed that phospho-c-Jun and GLS were both elevated downstream of the JNK constructs (Fig. 3g). Similarly, ectopic expression of the *JUN* gene resulted in upregulated *GLS* transcript levels (Fig. 3h), and elevated levels of both phospho-c-Jun and GLS (Fig. 3i).

Collectively, these results indicate that, downstream of oncogenic Rho GTPase signalling, JNK transmits a signal to increase transcription of the *GLS* gene by phosphorylating and activating the oncogenic transcription factor c-Jun. This prompted us to investigate the possible role of c-Jun in regulating *GLS* expression in human cancer cells.

### c-Jun and GLS correlate in human breast cancer cell lines

**Figure 4:**
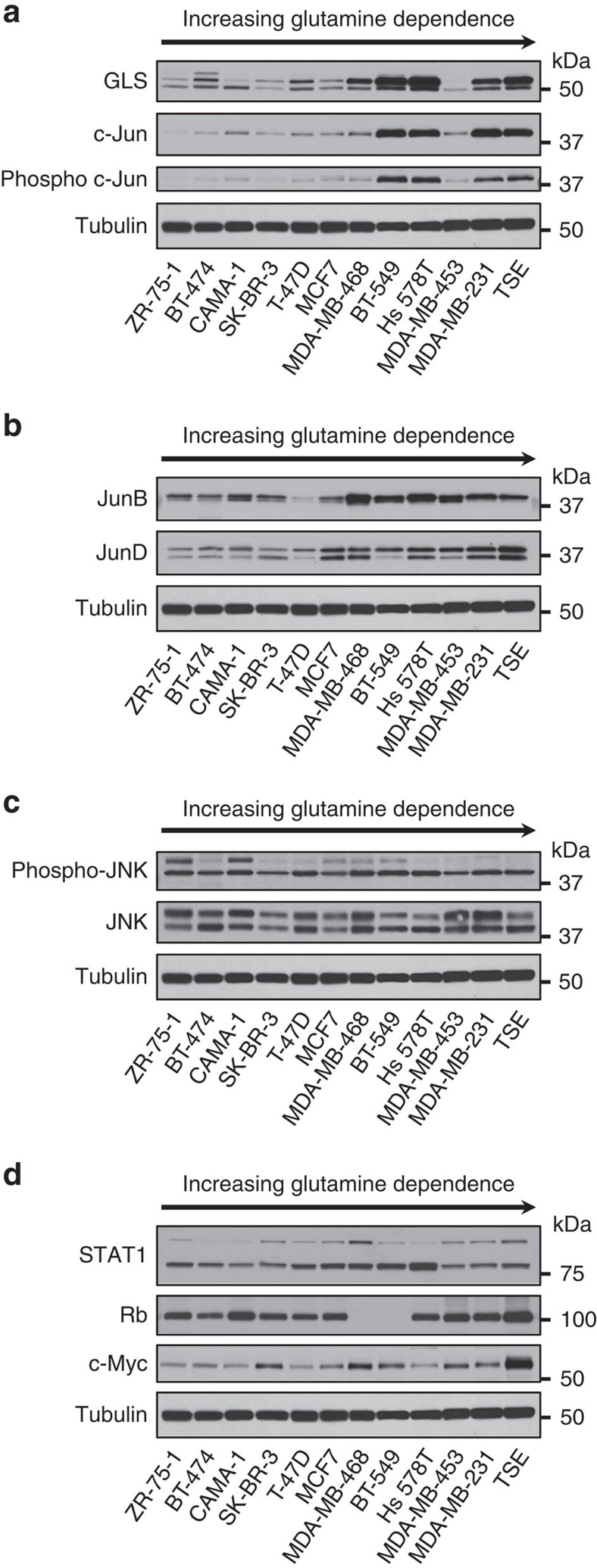
c-Jun correlates strongly with GLS levels in human breast cancer cell lines. Cells were collected at ∼60% confluency from RPMI growth medium supplemented with 10% FBS, and whole-cell lysates prepared and analysed by western blot. Samples were ordered according to glutamine dependence, increasing from left to right (Supplementary Fig. 3). (**a**) Correlation between c-Jun/phospho-c-Jun and GLS levels. Quantification of GLS and c-Jun band intensities allowed a Pearson correlation coefficient of 0.85 to be determined (Supplementary Fig. 4a). (**b**) Other Jun-family members do not correlate strongly with GLS levels. (**c**) Under 10% FBS culture conditions, p46 JNK (lower band) is active in all of the breast cancer cell lines. Neither JNK nor phospho-JNK correlate with GLS levels. (**d**) Other reported regulators of *GLS* expression do not strongly correlate with GLS levels.

Because the *JUN* proto-oncogene is overexpressed, and associated with aggressive behaviour, in a subset of human breast cancers^35^,^36^,^37^, we probed a panel of 12 breast cancer cell lines for c-Jun and GLS. First, we obtained an estimate of the glutamine dependence of each cell line by assaying proliferation over 6 days in culture medium containing either 2.0 or 0.1 mM glutamine (Supplementary Fig. 3). We then collected cells at ∼60% confluency from complete RPMI medium (10% FBS, 2 mM glutamine) and probed whole-cell lysates by western blot, with samples ordered by increasing glutamine dependence of the cell line, left to right. This revealed a very strong correlation (*R*=0.85) between relative levels of c-Jun and GLS (Fig. 4a and Supplementary Fig. 4a). In particular, cell lines with high endogenous c-Jun levels (BT-549, Hs 578T, MDA-MB-231 and TSE) all showed highly elevated GLS levels. The level of c-Jun also correlated very strongly with the glutamine dependence of the cell lines (*R*=0.83; see Supplementary Fig. 4b) and moderately strongly with their proliferation rates (*R*=0.63; see Supplementary Fig. 4c). The abundance of activated c-Jun, as indicated by phosphorylation at residue S73 (a JNK target residue), similarly correlated very strongly with GLS (Fig. 4a), whereas the related Jun-family transcription factors, JunB and JunD, showed less correlation (Fig. 4b). Relative levels of glutamate dehydrogenase (GLUD1/2) showed little variation among the cell lines (Supplementary Fig. 5).

The JNK1 and JNK2 proteins each exist as two splice variants, p46 (predominantly JNK1) and p54 (predominantly JNK2)^30^,^38^,^39^. Whereas p46 JNK1 is the primary kinase for activating c-Jun, endogenous p54 JNK2 can negatively regulate c-Jun stability^30^. We found that p46 JNK is expressed and phosphorylated to a similar extent across the panel of breast cancer cell lines (Fig. 4c). In contrast, although p54 JNK is expressed in all cell lines, it only shows strong phosphorylation in ZR-75-1 and CAMA-1 (Fig. 4c). Given that p46 JNK is activated in all cell lines, and that phosphorylated c-Jun is proportional to total c-Jun across the panel (Fig. 4a), we conclude that differences in total c-Jun activity between the cell lines are due primarily to variations in c-Jun protein levels, rather than to differences in JNK activity.

We also probed lysates for three previously reported regulators of GLS, namely c-Myc and STAT1 that upregulate GLS^22^,^23^,^40^, and the retinoblastoma protein (Rb), loss of which leads to elevated GLS levels through an unknown mechanism^41^ (Fig. 4d). None of these proteins correlated with GLS, although we note that all three are subject to post-translational regulation.

### The *GLS* promoter directly binds c-Jun at a consensus motif

We next examined whether c-Jun binds directly to the *GLS* promoter. The promoter region for the human *GLS* gene was analysed to position −5,000 bp relative to the transcription start site (TSS), and a number of putative c-Jun binding sites (<15% dissimilarity to the consensus sequence) were identified using the PROMO^42^ resource (Supplementary Fig. 6). An earlier genome-wide survey of c-Jun binding sites found them to be located in close proximity to the TSS, and mammalian transcription factor binding sites in general are strongly enriched around −200 bp from the TSS^35^,^43^. We found a close match to the consensus c-Jun binding motif (TGA[G/C]TCA) at position −188 bp relative to the TSS of human *GLS* (5′-TGACTCC-3′) (Supplementary Figs 6 and 7a). A close match to the c-Jun consensus motif is also present at position −200 bp in the mouse *GLS* promoter (5′-TGACACA-3′) (Supplementary Fig. 7b). Exact matches to the c-Jun consensus motif (5′-TGAGTCA-3′) were also found at position −2,211 bp for human *GLS*, and positions −2,469 and −1,545 bp for mouse *GLS*.

**Figure 5:**
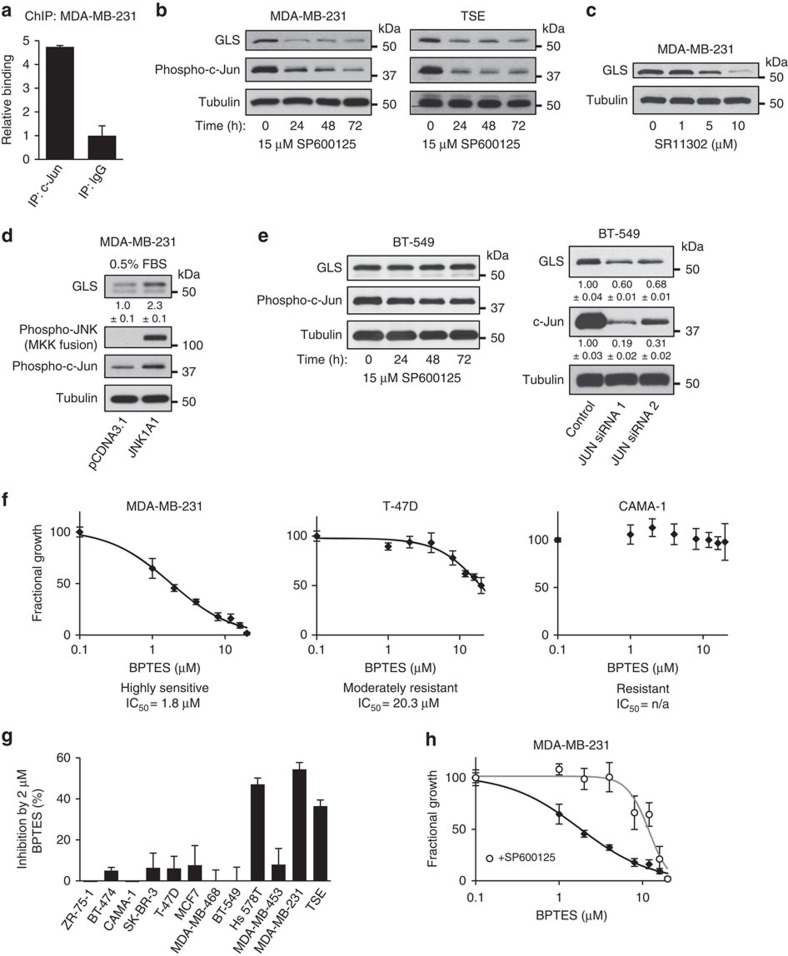
Inhibition of c-Jun suppresses *GLS* expression and BPTES sensitivity in human breast cancer cell lines. (**a**) ChIP analysis showing that c-Jun binds to the *GLS* promoter. Complexes containing c-Jun were immunoprecipitated from cross-linked, digested, chromatin isolated from MDA-MB-231 cells. A parallel immunoprecipitation using rabbit IgG was carried out as a negative control. Following reversal of cross-links and purification of DNA, RT–PCR was run using primers designed to amplify a 196-bp fragment centred on the putative c-Jun binding site at position −188 bp relative to the TSS. The data presented are the RQ values, with error bars marking RQ max and RQ min, from triplicate reactions. (**b**) Western blot analysis showing that treatment of MDA-MB-231 or TSE cells with the JNK inhibitor SP600125 (15 μM) leads to decreased phosphorylation of c-Jun, and decreased GLS levels. (**c**) Treatment of MDA-MB-231 cells with the AP-1 inhibitor SR11302 (1–10 μM) for 48 h results in a dose-dependent decrease in GLS. (**d**) Western blot analysis showing that transient transfection of MDA-MB-231 cells with a constitutively activated JNK fusion construct results in increased c-Jun phosphorylation and upregulated GLS levels. Cells were collected 48 h after transfection. (**e**) Western blot analysis showing that in the drug-resistant breast cancer cell line BT-549, treatment with 15 μM SP600125 has little effect on c-Jun phosphorylation and does not lead to decreased GLS levels (left panels). However, knockdown of *JUN* expression using siRNAs leads to decreased GLS levels. Relative band intensities are indicated. (**f**) Representative BPTES dose curves showing the effect of BPTES on the proliferation of breast cancer cell lines over 6 days. Curves were fitted using SigmaPlot, with data from triplicate assays. (**g**) Sensitivity of breast cancer cell lines to GLS inhibition, as indicated by inhibition of proliferation over 6 days by 2 μM BPTES. Of the high-c-Jun lines, only the drug-resistant BT-549 cells were not highly sensitive to BPTES. None of the low-c-Jun lines were highly sensitive. Data presented are the mean±s.d. of triplicate assays. (**h**) BPTES dose curves for MDA-MB-231 cells±15 μM SP600125, showing that inhibition of JNK desensitizes cells to GLS inhibition (the IC_50_ for BPTES shifts from 1.8 to 12 μM). Curves were fitted using SigmaPlot, with data from triplicate assays. Relative densitometry data are the mean±s.d. of triplicate blots.

We then carried out chromatin immunoprecipitations (ChIPs) to test whether c-Jun binds directly to the *GLS* promoter. For these experiments, we used MDA-MB-231 breast cancer cells, which have high endogenous levels of both GLS and c-Jun (Fig. 4a). Briefly, cross-linked chromatin was digested to a length of ∼150–900 bp, and an antibody against endogenous c-Jun was used to immunoprecipitate c-Jun/DNA complexes. A parallel immunoprecipitation was carried out using IgG, as a negative control. Protein-DNA cross-links were then reversed, and RT–PCR was performed using primers designed to amplify a 196-bp fragment centred on the putative c-Jun binding site at position −188 bp relative to the TSS (Supplementary Fig. 7a). This yielded a strong signal from the c-Jun ChIP relative to the IgG ChIP, indicating that c-Jun binds to this region of the *GLS* promoter (Fig. 5a). Similar results were obtained using two additional sets of primers to amplify slightly shorter fragments also centred on the predicted c-Jun binding site (Supplementary Fig. 8).

### c-Jun regulates *GLS* expression and BPTES sensitivity

Since c-Jun directly binds the promoter of the *GLS* gene, and endogenous levels of c-Jun and GLS correlate strongly in human breast cancer cell lines, we determined whether inhibition of JNK, inhibition of AP-1 family transcription factors, or knockdowns of c-Jun, affected GLS levels in these cells. We first inhibited JNK using 15 μM SP600125 in the high-GLS cell lines MDA-MB-231 and TSE. This led to a sharp decrease in phospho-c-Jun levels, and *GLS* expression was strongly suppressed within 24 h and remained so through 72 h (Fig. 5b). We also directly inhibited AP-1 transcriptional activity in MDA-MB-231 cells using SR11302 (48 h), and observed a dose-dependent suppression of *GLS* expression (Fig. 5c). Reciprocally, ectopic expression of a constitutively activated JNK1 fusion protein in MDA-MB-231 cells, under low serum (0.5% FBS) conditions, resulted in elevated c-Jun phosphorylation and elevated GLS levels (Fig. 5d).

In BT-549 cells, which are reported to be relatively drug-resistant^44^, treatment with 15 μM SP600125 caused only a very modest inhibition of c-Jun phosphorylation (Fig. 5e, left panel). Consistent with this, SP600125 treatment did not lead to decreased GLS levels in this cell line, and we therefore used siRNAs to knockdown *JUN* expression. Prolonged depletion of c-Jun in BT-549 cells (and in other cell lines with high endogenous c-Jun levels) could not be tolerated and resulted in cell death. Nevertheless, whole-cell lysates from BT-549 cells treated with *JUN*-targeted siRNAs and collected after 48 h repeatedly showed that GLS levels were lower in c-Jun-depleted cells (Fig. 5e, right panel). Quantification of band intensities confirmed that the degree of c-Jun depletion correlated with the degree of GLS depletion.

The results above indicate that the relationship between c-Jun and GLS levels in breast cancer cells is not only correlative but is also causative. We therefore examined whether cell lines with high c-Jun levels are more sensitive to the GLS inhibitor BPTES. Proliferation assays were carried out for all 12 breast cancer cell lines under a range of BPTES concentrations. Defining highly sensitive cell lines as those with an IC_50_<10 μM BPTES, none of the eight cell lines with low c-Jun levels were highly sensitive to BPTES, whereas three of the four cell lines with high c-Jun levels were highly sensitive (Supplementary Table 1). The one exception was the drug-resistant cell line BT-549. Representative dose curves for a highly sensitive cell line (MDA-MB-231, IC_50_=1.8 μM), a moderately resistant cell line (T-47D, IC_50_=20 μM), and a highly resistant cell line (CAMA-1, IC_50_=n/a) are shown (Fig. 5f). The much greater BPTES sensitivity of the high c-Jun cell lines, Hs 578T, MDA-MB-231 and TSE, relative to the low c-Jun lines, is illustrated by the response to 2 μM BPTES (Fig. 5g and Supplementary Fig. 9).

Inhibition of JNK by SP600125 led to a sharp decrease in GLS levels in MDA-MB-231 (and other) breast cancer cells (see above). We therefore carried out proliferation assays to compare BPTES sensitivity of MDA-MB-231 cells cultured in the absence or presence of 15 μM SP600125. Treatment with the JNK inhibitor sharply decreases the proliferation rate relative to untreated cells. However, the slow proliferation that still occurs in the presence of SP600125 is markedly desensitized to BPTES, with the IC_50_ for BPTES shifting ∼7-fold from 1.8 to 12 μM (Fig. 5h).

### c-Jun is sufficient to elevate GLS in breast cancer cells

**Figure 6:**
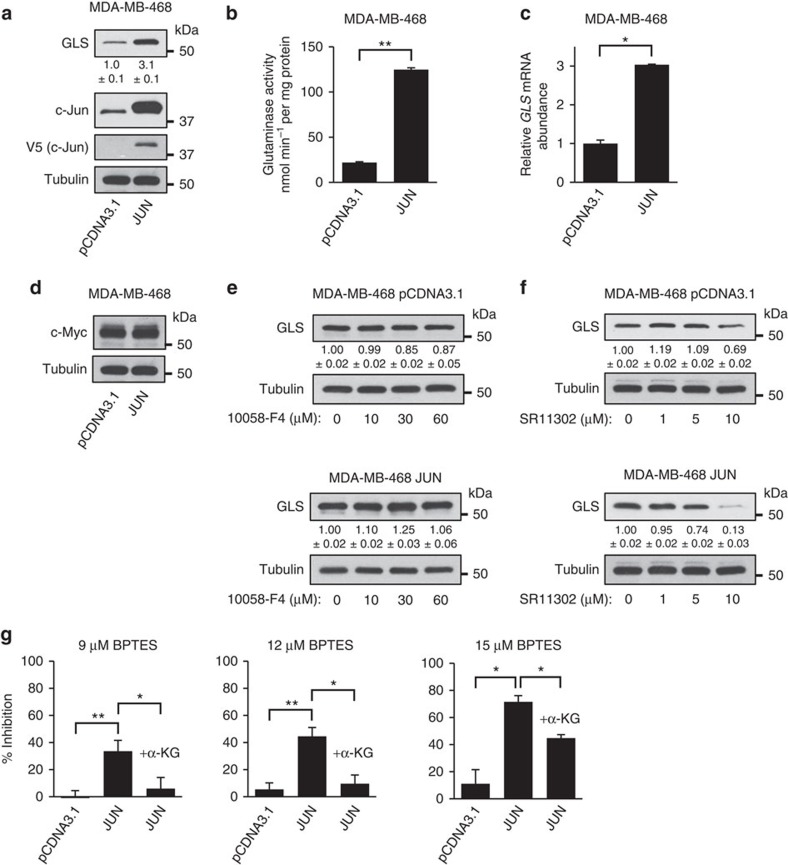
c-Jun increases *GLS* expression and BPTES sensitivity in breast cancer cells. (**a**) Western blot analysis of GLS and c-Jun levels in whole-cell lysates of MDA-MB-468 cells, stably carrying either pCDNA3.1 or the *JUN-V5* expression vector pCDNA3.1-JUN. Relative band intensities are indicated for GLS. (**b**) Glutaminase activity assay using mitochondria isolated from the derivative MDA-MB-468 cell lines. Activity is expressed per mg of total cellular protein, and data presented are the mean±s.d. of triplicate assays. (**c**) RT–PCR analysis of the derivative MDA-MB-468 cell lines, showing relative abundance of the *GLS* transcript. The data presented are the RQ values, with error bars marking RQ max and RQ min, from triplicate reactions. (**d**) Western blot analysis showing c-Myc levels in the derivative cell lines. (**e**) Western blot analysis showing that treatment of vector-control, or *JUN*-overexpressing, MDA-MB-468 cells with the c-Myc inhibitor 10058-F4 at concentrations up to 60 μM for 48 h has little effect on GLS levels. Relative band intensities are indicated for GLS. (**f**) Western blot analysis showing that treatment with the AP-1 inhibitor SR11302 (1–10 μM) for 48 h leads to a dose-dependent decrease in GLS levels. The effect is especially pronounced in *JUN*-overexpressing cells. (**g**) Cell proliferation assays for MDA-MB-468 cells stably carrying pCDNA3.1 or the *JUN* expression vector. Cells were seeded in 12-well dishes at a density of 1 × 10^4^ cells per well, cultured for 6 days in the absence or presence of 9 μM (left panel), 12 μM (middle panel) or 15 μM (right panel) BPTES±1 mM dimethyl α-KG. Inhibition of proliferation by BPTES treatment is plotted as a percentage. The data presented are the mean±s.d. of triplicate assays. Relative densitometry data are the mean±s.d. of triplicate blots. Differences were analysed with Student's *t*-test. *P<0.05, ^**^P<0.01.

Using the MDA-MB-468 breast cancer cell line, which exhibits relatively low endogenous levels of c-Jun and moderate levels of GLS (Fig. 4a), we generated derivative cell lines that stably overexpress *JUN-V5* or carry the empty plasmid vector. We then collected vector-control cells and *JUN*-overexpressing cells at ∼60% confluency from RPMI medium (10% FBS, 2 mM glutamine), and probed whole-cell lysates for GLS, c-Jun and V5-tag by western blot (Fig. 6a). This showed that GLS levels are elevated in the *JUN*-overexpressing cells relative to the vector-control cells. Mitochondria isolated from the two cell lines were assayed for glutaminase activity, which was markedly higher in the *JUN*-overexpressing cells (Fig. 6b). Moreover, RT–PCR analysis confirmed that *GLS* transcript levels were upregulated in *JUN*-overexpressing cells relative to vector-control cells (Fig. 6c). We made equivalent derivative cell lines of the MCF7 parental cell line, which has very low endogenous levels of both c-Jun and GLS (Fig. 4a), and similarly found that *GLS* expression was upregulated in *JUN*-overexpressing cells (Supplementary Fig. 10a).

The level of c-Myc did not vary between the vector-control and *JUN*-overexpressing cells (Fig. 6d), and treatment with the c-Myc inhibitor 10058-F4 at concentrations up to 60 μM (48 h) had little effect on GLS levels in the derivative cell lines (Fig. 6e and Supplementary Fig. 10b). In contrast, treatment with the AP-1 inhibitor SR11302 (1–10 μM) caused a dose-dependent suppression of GLS in both cell lines, an effect that was especially pronounced in the *JUN*-overexpressing cells (Fig. 6f).

### Overexpression of *JUN* sensitizes cancer cells to BPTES

As described above, MDA-MB-231 cells treated with the JNK inhibitor SP600125 become desensitized to the GLS inhibitor BPTES (Fig. 5h). To complement this result, we carried out proliferation assays to determine whether overexpression of the *JUN* proto-oncogene is sufficient to increase the BPTES sensitivity of MDA-MB-468 cells. Although vector-control cells showed some response to BPTES treatment, overexpression of *JUN* greatly sensitized cells to BPTES treatment (Fig. 6g). Supplementation of the medium with 1 mM dimethyl α-KG completely rescued cells from ≤12 μM BPTES, and partially rescued cells from higher BPTES concentrations (Fig. 6g). Although the parental and vector-control MDA-MB-468 cell lines showed little dependence on the GLS reaction, both are moderately glutamine-dependent (Supplementary Figs 3 and 11). This reflects the importance of an exogenous glutamine supply for other metabolic processes that do not involve the GLS reaction, such as nucleotide biosynthesis and protein synthesis. Glutamine dependence of the *JUN*-overexpressing derivative cell line was unchanged from that of the parental cells (Supplementary Fig. 11).

### c-Jun regulated transcript levels in invasive breast cancer

**Figure 7:**
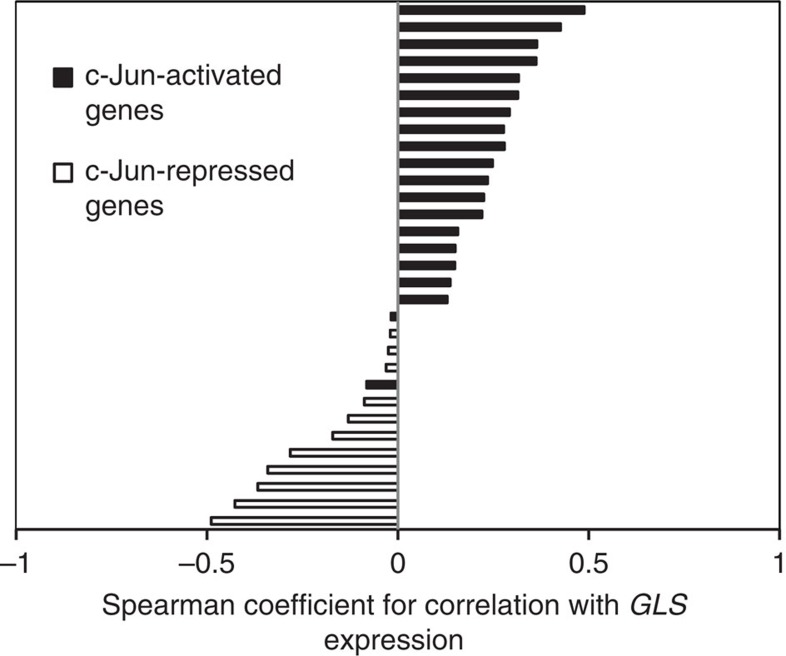
The *GLS* transcript correlates with established c-Jun target transcripts in invasive breast cancer. Bar chart showing the Spearman correlation coefficients between mRNA levels of *GLS* and of established c-Jun transcriptional targets. Transcripts that are upregulated by c-Jun are shown in black, and almost exclusively correlate positively with *GLS*. Transcripts that are repressed by c-Jun are shown in white, and all correlate negatively with *GLS*. Correlation plots were prepared and correlation coefficients determined using the cBioportal suite of tools and data from The Cancer Genome Atlas (TCGA) Breast Invasive Carcinoma (TCGA, provisional) data set.

To establish further the relationship between c-Jun activity and *GLS* expression in breast cancer, we used the cBioportal^45^,^46^ suite of tools (www.cbioportal.org) to analyse data from The Cancer Genome Atlas (TCGA) Breast Invasive Carcinoma (TCGA, provisional) data set (959 samples). In many cancers, upregulation of c-Jun occurs at the post-transcriptional and post-translational levels, but not at the transcriptional level (see Discussion). Consequently, *JUN* mRNA levels are rarely upregulated, even though c-Jun protein levels can be highly elevated. Consistent with these findings, TCGA data showed that *JUN* mRNA was elevated (*z* score >2.0) in only 3% of invasive breast carcinoma samples. We therefore determined whether the expression levels of some validated c-Jun transcriptional targets correlate with those of *GLS*. We selected only confirmed direct targets of c-Jun (and not v-Jun) that contain a c-Jun binding site within the promoter region^35^,^47^,^48^,^49^, and we eliminated from the list those c-Jun target genes that are frequently altered at the genomic level within the data set (for example, *CCND1*, amplified in 16% of samples, and *TP53*, mutated in 31% of samples). Using the resulting list of c-Jun targets (Supplementary Table 2), we found that transcripts that are upregulated by c-Jun almost invariably correlate positively with *GLS* mRNA levels, whereas those targets that are transcriptionally repressed by c-Jun exhibit a negative correlation with the *GLS* transcript (Fig. 7; see also Supplementary Figs 12 and 13).

## Discussion

In proliferating cells, the TCA cycle serves as a major source of biosynthetic precursors. Metabolic intermediates that are lost to anabolic pathways must be rapidly replenished, and the most abundant carbon sources for these anaplerotic reactions are glucose and glutamine. Indeed, anaplerosis is a key function underlying the upregulated consumption of these nutrients by cancer cells^10^,^11^,^50^. An important route for the delivery of glutamine-derived carbon into the TCA cycle begins with the hydrolysis of glutamine to glutamate, a reaction catalysed by glutaminase. The TCA cycle intermediate α-ketoglutarate can then be generated through the action of glutamate dehydrogenase, or through transaminase activities.

In this study, we used an inducible MEF system for hyper-activating Rho GTPase signalling, which we previously linked to increased mitochondrial GLS activity in NIH-3T3 cells^20^, and also to upregulated glutamine-mediated anaplerosis for oxidative TCA cycle flux in MEFs^51^. Ectopic expression of oncogenic-Dbl in NIH-3T3 cells leads to elevated levels of the *GLS* transcript (Supplementary Fig. 14a), but the lower background level of GLS in MEFs relative to NIH-3T3 cells (Supplementary Fig. 14b) allows for a much clearer read-out, by RT–PCR and especially by western blot, of changes in *GLS* expression. By selectively inhibiting signalling pathways activated downstream of the Rho GTPases in the inducible system, we have now discovered that the c-Jun N-terminal kinase (JNK) and the oncogenic transcription factor c-Jun are critical for upregulating GLS at the transcript and protein levels. We also found that in human breast cancer cell lines, c-Jun levels correlate strongly with GLS levels and with sensitivity to the GLS inhibitor BPTES. Moreover, we determined that c-Jun directly binds the promoter of the *GLS* gene and increases its expression. Importantly, this not only leads to elevated mitochondrial glutaminase activity, but also causes cancer cells to become more dependent on glutamine-mediated anaplerosis.

The c-Jun transcription factor is the cellular homologue of v-Jun, the transforming oncoprotein of avian sarcoma virus 17 (refs 52, 53). A key role for c-Jun in both healthy and neoplastic tissue is to drive cell cycle progression, and fibroblasts derived from *JUN*-null mouse embryos exhibit a severe defect in proliferation^54^. The c-Jun protein is phosphorylated at the M/G1 transition^55^, and loss of c-Jun or expression of mutants defective for JNK-catalysed phosphorylation leads to a G2/M cell cycle block^56^. One transcriptional target of c-Jun is the *CCND1* gene, which encodes the G1 to S phase regulator cyclin D1 (ref. 57). c-Jun also binds to a variant AP-1 site in the *TP53* gene promoter, leading to suppression of p53 and of the p53-regulated cyclin-dependent kinase inhibitor p21 (ref. 54). The net effect is that c-Jun increases the activity of G1 cyclin-dependent kinase complexes.

**Figure 8:**
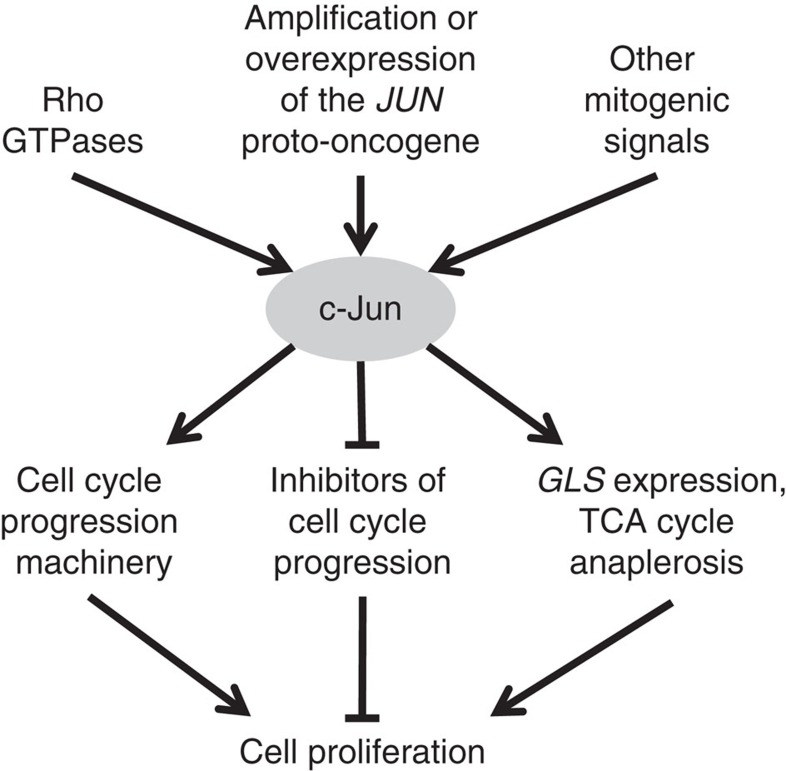
c-Jun coordinates cell cycle progression with metabolic reprogramming. Diagram summarizes the coordinated regulation of cell proliferation by c-Jun. Transcriptional activity of c-Jun can be enhanced by activating phosphorylations downstream of mitogenic signalling pathways, including those mediated by the Rho family of GTPases. c-Jun can also be upregulated by amplification or increased expression of the *JUN* proto-oncogene. A key role for c-Jun is to drive cell cycle progression, which is achieved through transcriptional repression of cell cycle inhibitors and transcriptional activation of cell cycle progression machinery. In this study, we report that c-Jun also controls metabolic reprogramming to support proliferation, by upregulating *GLS* expression and thereby stimulating delivery of glutamine-derived carbon into the TCA cycle.

When taken together, these findings provide a biological rationale for the positive regulation of GLS by c-Jun that we describe. In order for sustained proliferation to occur, signalling to the cell cycle machinery must be coordinated with reprogramming of cellular metabolism to support biomass accumulation. By simultaneously driving cell cycle progression and upregulating *GLS* expression, c-Jun promotes cell proliferation and also activates TCA cycle anaplerosis to replenish metabolites that have been directed to biosynthetic pathways (Fig. 8). Notably, two other reported regulators of *GLS* expression are involved in cell cycle control. The Rb protein prevents G1 to S phase progression^58^, and loss of this tumour suppressor leads to upregulation of GLS^41^. Similarly, the oncogenic transcription factor c-Myc promotes G1 to S phase transition, and is a positive regulator of *GLS* expression^22^,^23^.

Mirroring the ability of v-Jun to cause avian sarcoma, the *JUN* gene is highly amplified and overexpressed in aggressive human sarcomas^59^, and in an analysis of copy number alterations across 3,131 diverse cancer samples, *JUN* was found to be significantly amplified across the entire data set^60^. In many cancers, protein levels of c-Jun are highly upregulated even in the absence of *JUN* amplifications or elevated transcription of the *JUN* gene. This can be due to both translational activation and increased protein stability. For example, c-Jun is highly elevated in melanoma cells as a result of increased translation following loss of mi-R125b, which binds to the coding region of the *JUN* transcript and suppresses translation in normal melanocytes^61^. In breast cancer cells, c-Jun is also upregulated at the protein level but not at the transcriptional level^37^. One mechanism for this involves downregulation of the tumour suppressor COP1 E3 ubiquitin ligase, which targets c-Jun for degradation^37^. Indeed, among the cell lines used in the present study, BT-549, Hs 578T and MDA-MB-231 are reported to have low COP1 levels, whereas MCF7 and T47D have elevated COP1 (ref. 37).

For transcriptional activity, c-Jun and related transcription factors must form a dimeric complex, which is known as AP-1 and consists of Jun–Jun or Jun–Fos dimers. Jun-family members (c-Jun, JunB and JunD) can dimerize with themselves or with Fos proteins, whereas Fos-family members (c-Fos, Fra-1, Fra-2 and FosB) must heterodimerize with a Jun protein^57^. This raises some questions concerning the fine-tuning of *GLS* regulation. Of the AP-1 transcription factors, c-Jun is the most potent transcriptional activator. However, Jun-Fos heterodimers are more stable and efficient at driving transcriptional activation than Jun–Jun dimers^57^. Importantly, the promoter specificity of dimers containing c-Jun differs according to the dimerization partner, and the relative abundances of potential partners vary between cell types^62^,^63^. Consequently, the Jun- and Fos-family members that are expressed within a given cell likely influence the regulation of *GLS* expression by c-Jun, and it is possible that JunB or JunD might substitute for c-Jun in some contexts.

High levels of c-Jun are associated with aggressive, invasive and metastatic behaviour in breast cancer. Ectopic overexpression of *JUN* in the MCF7 cell line leads to increased invasion and tumorigenicity, whereas a dominant-negative variant of c-Jun causes cell cycle arrest^64^,^65^. Several studies have found that c-Jun is specifically elevated in triple-negative breast cancer (TNBC) cells^35^,^37^,^66^ and *MYC* expression is also reported to be upregulated in TNBC^67^. This could explain why TNBC cell lines have higher GLS levels and greater sensitivity to the GLS inhibitor CB-839 (a BPTES derivative) than receptor-positive breast cancer cells^21^.

In conclusion, we have identified a JNK/c-Jun-dependent signalling pathway that is responsible for upregulating GLS levels during cellular transformation. Moreover, we show that the c-Jun transcription factor is an important regulator of *GLS* expression in breast cancer, and can drive cellular dependence on the glutaminase reaction, thus conferring sensitivity to the GLS inhibitor BPTES. Our findings link a key player in cell cycle progression with a metabolic pathway that supports cell proliferation, and illustrate that re-programming of cellular metabolism is intricately connected with oncogenic transformation. Furthermore, our work reveals a novel function for the c-Jun oncoprotein in cancer, and suggests why certain types of cancer such as triple-negative breast cancer might be more susceptible to glutaminase-targeted therapy.

## Methods

### Isogenic inducible MEF system for oncogenic-Dbl expression

The inducible MEF line used in this study was generated as described previously^51^. Briefly, the gene encoding oncogenic-Dbl was sub-cloned into vector pTRE-HA (Clontech). Parental MEFs containing the transcriptional transactivator tTA (Clontech) were then co-transfected with the resulting pTRE-HA-onco-Dbl vector along with vector pMET-puro, in a 20:1 ratio. Following puromycin selection (∼3 weeks), colonies were screened for inducible expression of oncogenic-Dbl. Cells were maintained at 37 °C, 5% CO_2_ atmosphere, in DMEM medium containing 4 mM glutamine (Gibco) and supplemented with 10% (v/v) tetracycline-free FBS (Gibco) and 0.6 μg ml^−1^ doxycycline. Expression of oncogenic-Dbl was induced by re-plating cells in doxycycline-free medium (10% FBS). Residual doxycycline was removed by replacing the medium (with the appropriate concentration of FBS, and small-molecule inhibitor where relevant) after 5 h. Uninduced control samples were treated in the same way: re-plating followed by media change at 5 h, but with 0.6 μg ml^−1^ doxycycline present at all times. For glutamine-withdrawal experiments, glutamine-free DMEM medium (Gibco) was supplemented with dialysed FBS (Gibco). Growth medium containing other concentrations of glutamine was prepared by mixing appropriate volumes of DMEM (4 mM glutamine) and glutamine-free DMEM.

### Breast cancer cell culture and media

Breast cancer cell lines ZR-75-1, BT-474, CAMA-1, SK-BR-3, T-47D, MCF7, MDA-MB-468, BT-549, Hs 578T, MDA-MB-453 and MDA-MB-231 were obtained from the American Type Cell Culture Collection (ATCC). The TSE breast cancer cell line was kindly supplied by Dr Steven Abcouwer (University of Michigan). All breast cancer cell lines were maintained at 37 °C, 5% CO_2_ atmosphere, in RPMI 1640 medium containing 2 mM glutamine (Gibco) and supplemented with 10% FBS (Gibco). For glutamine-withdrawal experiments, glutamine-free RPMI 1640 medium (Gibco) supplemented with 10% dialysed FBS (Gibco) was used. Cell lines were periodically tested for Mycoplasma contamination.

### DNA constructs

DNA primers were synthesized by Integrated DNA Technologies. Plasmid pCDNA3.1/V5-His TOPO was purchased from Life Technologies. The *JUN* gene was PCR amplified from plasmid pMIEG3-c-Jun, which was a gift from Alexander Dent (Addgene plasmid # 40348)^68^, using primers JUN_AMP_F (5′-TTATGGATCCATGACTGCAAAGATGGAAACGACC-3′) and JUN_AMP_R (5′-TTATGATATCAAATGTTTGCAACTGCTGCGTTAGC-3′), which added a BamH1 site and EcoRV site, respectively. The PCR product was purified (QIAquick PCR purification kit, QIAGEN), digested with BamH1 and EcoRV (New England Biolabs), re-purified and ligated (T4 DNA ligase, New England Biolabs) into pre-digested and purified pCDNA3.1/V5-His TOPO to yield an expression vector for *JUN-V5*, pCDNA3.1-JUN. The construct was verified by sequencing. The plasmids pCDNA3 Flag MKK7B2Jnk1a1 (Addgene plasmid # 19726)^34^, pCDNA3 Flag MKK7B2Jnk2a2 (Addgene plasmid # 19727)^34^ and pCDNA3 Flag MKK7B2Jnk3a2 (Addgene plasmid # 19729)^34^ were all gifts from Roger Davis.

### Antibodies and reagents

Antibodies recognizing the following proteins were purchased from Cell Signaling Technology: HA-tag (cat. no. 3724) used at 1:5,000, α/β-tubulin (cat. no. 2148) used at 1:5,000, phospho-SEK1/MKK4 (S257) (cat. no. 4514) used at 1:2,000, phospho-MKK7 (S271/T275) (cat. no. 4171) used at 1:2,000, phospho-JNK (T183/Y185) (cat. no. 4668) used at 1:2,000, phospho-c-Jun (S73) (cat. no. 3270) used at 1:2,000, c-Jun (cat. no. 9165) used at 1:2,000, JunB (cat. no. 3753) used at 1:2,000, JunD (cat. no. 5000) used at 1:2,000, phospho-p38 MAPK (T180/Y182) (cat. no. 4511) used at 1:2,000, p38 MAPK (cat. no. 8690) used at 1:2,000, phospho-ERK1/2 (T202/Y204) (cat. no. 4370) used at 1:2,000, ERK1/2 (cat. no. 9102) used at 1:2,000, phospho-myosin light chain 2 (S19) (cat. no. 3675) used at 1:1,000, myosin light chain 2 (cat. no. 3672) used at 1:1,000, phospho-AMPKα (T172) (cat. no. 4188) used at 1:2,000, AMPKα (cat. no. 2603) used at 1:2,000, phospho-Akt (T308) (cat. no. 9275) used at 1:2,000, Akt (cat. no. 9272) used at 1:2,000, phospho-p70 S6 kinase (T389) (cat. no. 9205) used at 1:2,000, p70 S6 kinase (cat. no. 9202) used at 1:2,000, STAT1 (cat. no. 9172) used at 1:2,000, Rb (cat. no. 9309) used at 1:2,000, Glut1 (cat. no. 12939) used at 1:4,000, and c-Myc (cat. no. 5605) used at 1:2,000. The antibody recognizing GLS was purchased from Abgent (cat. no. AP8809b) and used at 1:8,000. Clarification of the bands recognized by this antibody is shown in Supplementary Fig. 15. Antibodies recognizing MKK4 (MEK4) (cat. no. ab33912), MKK7 (MEK7) (cat. no. ab52618) and phospho-c-Myc (T58+S62) (cat. no. ab10568), all used at 1:4,000, were purchased from Abcam. Antibodies recognizing JNK (cat. no. sc-571) and GLUD1/2 (cat. no. sc-160383), both used at 1:2,000, were purchased from Santa Cruz Biotechnology. The antibody recognizing V5-tag was purchased from Life Technologies (cat. no. R960) and used at 1:5,000. Secondary antibodies used, as appropriate, were Cell Signaling Technology anti-rabbit IgG, HRP-linked (cat. no. 7074) or anti-mouse IgG, HRP-linked (cat. no. 7076), or Santa Cruz Biotechnology donkey anti-goat IgG-HRP (cat. no. sc-2020). BPTES was synthesized and kindly provided by Dr Scott Ulrich, Ithaca College. ROCK inhibitor Y-27632 (10 mM solution in DMSO), the JNK inhibitor SP600125 (50 mM solution in DMSO) and G418 were purchased from Calbiochem. All other reagents were purchased from Sigma-Aldrich unless otherwise stated.

### Western blot analysis

Whole-cell lysates were prepared in lysis buffer (50 mM HEPES pH 8.0, 150 mM NaCl, 1 mM Na_3_VO_4_, 25 mM NaF, 1% (v/v) Triton X-100, 1 mM MgCl_2_, 50 mM β-glycerophosphate, 30 μg ml^−1^ leupeptin, 5 μg ml^−1^ aprotinin) and insoluble debris was pelleted by centrifugation and removed. Protein concentration was determined by Bradford assay (Bio-Rad), and lysate proteins denatured by boiling for 5 min in reducing SDS-sample buffer. Lysate proteins (20 μg total protein/lane) were then resolved on Novex 4–20% Tris-glycine mini or midi protein gels (Life Technologies), and transferred to polyvinylidene difluoride membranes (PerkinElmer). Membranes were blocked in 7% bovine serum albumin in tris-buffered saline and tween 20 (TBST) for 1 h at room temperature, and probed overnight at 4 °C in primary antibody solution (manufacturer recommended concentration) in TBST. They were then washed in TBST, and incubated in TBST solution containing 25% (v/v) non-fat dry milk powder and appropriate secondary antibody at the manufacturer's recommended concentration for 1 h. Finally, the membranes were washed in TBST, and bands imaged using Western Lightning Plus-ECL (PerkinElmer) and HyBlot ES autoradiography film (Denville Scientific Inc.).

### MEF saturation density analysis

DMEM medium supplemented with 10% FBS±0.6 μg ml^−1^ doxycycline was added to 60 mm dishes (3 ml per dish), and dishes were then seeded with 1 × 10^5^ MEFs per dish. Following cell attachment (5 h), growth medium was replaced with fresh medium containing appropriate supplements. Growth medium was subsequently replaced at 48 h intervals. At day 8, growth medium was removed and 1.5 ml 3.7% formaldehyde solution in H_2_O was added for 30 min. The formaldehyde solution was removed, and 1.5 ml crystal violet solution was added for 20 min at room temperature. This was then removed, and dishes washed four times with 3 ml H_2_O and allowed to dry before imaging.

### MEF anchorage-independent growth assay

Uninduced or oncogenic-Dbl-induced MEFs were seeded at a density of 8,000 cells per ml in medium containing 0.3% (w/v) agarose, onto underlays of medium containing 0.6% agarose, in six-well plates. Both layers of medium were supplemented with appropriate concentrations of doxycycline (uninduced cells) and BPTES. Cultures were fed every 3 days, and the total number of colonies was counted after 15 days.

### Cell proliferation assays

Culture medium supplemented with glutamine or with inhibitors at the described concentrations was added to 12-well plates (1 ml per well), and wells were seeded with cells at day 0 as follows (numbers optimized to avoid over-confluence during the assay). TSE cells: 5 × 10^3^ cells per well. MDA-MB-231, MDA-MB-453, MDA-MB-468, BT-549: 1 × 10^4^ cells per well. Hs 578T, MCF7, T-47D, CAMA-1, SK-BR-3, BT-474: 1.5 × 10^4^ cells per well. ZR-75-1: 2 × 10^4^ cells per well. Following cell attachment, growth medium was replaced at 12 h and subsequently every 48 h. At day 6, cells were trypsinized, suspended in an appropriate volume of medium, and a hemacytometer was used to determine the total number of cells per well. All cell proliferation assays were carried out in triplicate, and the mean and s.d. calculated.

### Mitochondrial isolation

Mitochondria were isolated using the Qproteome mitochondria isolation kit (QIAGEN), following the manufacturer's instructions. For MEFs grown in low-serum media, 3 × 15 cm dishes of cells at ∼70% confluency were collected, and for MDA-MB-468 cells, 2 × 15 cm dishes of cells at ∼70% confluency were collected. The total protein of the starting material (collected cells) and of the isolated mitochondria suspension was measured by Bradford assay (Bio-Rad). This allowed glutaminase activity to be determined per unit of total cellular protein (see below).

### Mitochondrial glutaminase activity assay

A two-step protocol was used to assay glutaminase activity, as previously described^28^. In the first reaction, glutaminase catalyses the hydrolysis of glutamine to glutamate, and in the second reaction glutamate dehydrogenase catalyses the oxidative deamination of glutamate to form α-ketoglutarate and NADH, which can be measured through its absorbance at 340 nm. Briefly, mitochondria (10 μg total protein) were added to 105 μl of reaction mix 1 (20 mM glutamine, 0.2 mM EDTA, 50 mM Tris-acetate, pH 8.6). Samples were rotated at 37 °C for 45 min. The reaction was then quenched by adding 10 μl of 3 M HCl, and samples were placed on ice. Next, 20 μl of reaction mix 1 was added to 200 μl of reaction mix 2 (1 unit bovine liver glutamate dehydrogenase (Sigma-Aldrich), 80 mM Tris-HCl pH 9.4, 200 mM hydrazine, 0.25 mM ADP, 2 mM NAD). The samples were mixed and incubated for 1 h at room temperature. The A_340_ was then determined against a blank in which a heat-inactivated mitochondrial sample was added to reaction mix 1. A standard curve was also prepared by adding known amounts of glutamate to reaction 2. This allowed the amount of glutamate generated during reaction 1 to be determined. All assays were carried out in triplicate, and the mean and s.d. calculated.

### Real-time PCR analysis

Total RNA was extracted from cells using the RNeasy mini kit (QIAGEN), and cDNAs were prepared using the SuperScript III first-strand synthesis system (Life Technologies). RT-PCR was carried out using the 7,500 fast real-time PCR system (Applied Biosystems), with cDNA as a template and *GLS* primers GLS-F (5′-TGTCACGATCTTGTTTCTCTGTG-3′) and GLS-R (5′-TCATAGTCCAATGGTCCAAAG-3′). Primers for mouse *GAPDH* (mGAPDH-F, 5′-ACAGTCCATGCCATCACTGCC-3′ and mGAPDH-R, 5′-GCCTGCTTCACCACCTTCTTG-3′) or human *ACTB* (hACTB-F, 5′-CATCGAGCACGGCATCGTCA-3′ and hACTB-R, 5′-TAGCACAGCCTGGATAGCAAC-3′) were used as endogenous controls, as appropriate. All reactions were carried out using *POWER* SYBR green PCR master mix (Life Technologies). At least three replicates of each PCR were carried out.

### Chromatin immunoprecipitation

Chromatin immunoprecipitations were performed using the SimpleChIP enzymatic chromatin IP kit (Cell Signaling Technology), following the manufacturer's instructions. MDA-MB-231 cells (5 × 15 cm dishes at ∼85% confluency) were used as the source of chromatin. Analysis following chromatin digestion showed that DNA was digested to fragments of the desired size (150–900 bp, equivalent to 1–5 nucleosomes). An antibody against endogenous c-Jun (Cell Signaling Technology, 9165) was used to immunoprecipitate complexes containing c-Jun. Following reversal of protein–DNA complexes and purification of DNA, RT–PCR was carried out as described above but using the purified DNA as a template. Three primer sets were designed to amplify 100–200 bp fragments centred on the putative c-Jun binding site of the *GLS* promoter at position −188 bp relative to the TSS. Set 1 (forward and reverse): 5′-CCCTAGTACCCAACTAGGCTAGCC-3′ and 5′-CCTCTCTTTTGATTGGCGATTAGGG-3′. Set 2 (forward and reverse): 5′-GCGTGCAGAAAGTGGCTACTGAGC-3′ and 5′-CTCTCGGCTCTGGGTGCGCGGAGAG-3′. Set 3 (forward and reverse): 5′-CCTCGGAGTTGGCACGGCGTGCAG-3′ and 5′-GGCAGTCAAATTTCTCTCGGCTC-3′.

### RNA interference

Knockdown of *JUN* expression in BT-549 cells was achieved using Silencer select pre-designed and validated siRNA (Life Technologies). Because endogenous c-Jun levels are so high in this cell line, two rounds of siRNA treatment were required to achieve effective knockdowns. Two siRNA products (manufacturer ID # s7658 and s7659) were used, along with the Silencer select negative control no. 1 siRNA. Transfections were carried out in 10 cm dishes at ∼80% confluency using Lipofectamine 2000 transfection reagent (Life Technologies) and following the manufacturer's instructions. The final siRNA concentration was 15 nM in all cases. Transfection medium was replaced with fresh growth medium 5 h after treatment. At 48 h following the initial transfection, the procedure was repeated, and cells were collected after a further 48 h.

### Stable cell lines

Transfection of breast cancer cell lines with plasmid DNA constructs (pCDNA3.1 or pCDNA3.1-JUN) was carried out using Lipofectamine 2000 transfection reagent (Life Technologies), following the manufacturer's instructions. At 48 h after transfection, cells were placed under G418 selection by supplementing the growth medium with G418 (500 μg ml^−1^ for MCF7, and 800 μg ml^−1^ for MDA-MB-468). Growth medium was replaced every 48 h for 2–3 weeks, until isolated colonies (∼2 mm diameter) were apparent on the plate. At this point, individual clones were transferred to 12-well dishes and expanded in 250 μg ml^−1^ G418 for further analysis. Appropriate clones were screened for c-Jun-V5 by western blot, and V5-positive clones were maintained in 250 μg ml^−1^ G418.

### TCGA data

The Cancer Genome Atlas (TCGA) Breast Invasive Carcinoma (TCGA, provisional) data set was accessed, all data were analysed, and correlation plots prepared using the cBioportal^45^,^46^ suite of tools (www.cbioportal.org).

### Statistical analyses

All differences were analysed with Student's *t*-test. A *P* value <0.05 was considered to be significant and marked (*), and a *P* value below 0.01 was considered to be highly significant and marked (**).

## 

## Additional information

**How to cite this article:** Lukey, M. J. *et al.* The oncogenic transcription factor c-Jun regulates glutaminase expression and sensitizes cells to glutaminase-targeted therapy. *Nat. Commun.* 7:11321 doi: 10.1038/ncomms11321 (2016).

## Supplementary Material

Supplementary Information Supplementary Figures 1-16 and Supplementary Tables 1-2

## References

[b1] DeBerardinis R. J., Lum J. J., Hatzivassiliou G. & Thompson C. B. The biology of cancer: metabolic reprogramming fuels cell growth and proliferation. Cell Metab. 7, 11–20 (2008). 18177721 10.1016/j.cmet.2007.10.002

[b2] Dang C. V. Links between metabolism and cancer. Genes Dev. 26, 877–890 (2012). 22549953 10.1101/gad.189365.112 PMC3347786

[b3] Lunt S. Y. & Vander Heiden M. G. Aerobic glycolysis: meeting the metabolic requirements of cell proliferation. Annu. Rev. Cell Dev. Biol. 27, 441–464 (2011). 21985671 10.1146/annurev-cellbio-092910-154237

[b4] Tong X., Zhao F. & Thompson C. B. The molecular determinants of de novo nucleotide biosynthesis in cancer cells. Curr. Opin. Genet. Dev. 19, 32–37 (2009). 19201187 10.1016/j.gde.2009.01.002 PMC2707261

[b5] Lukey M. J., Wilson K. F. & Cerione R. A. Therapeutic strategies impacting cancer cell glutamine metabolism. Future Med. Chem. 5, 1685–1700 (2013). 24047273 10.4155/fmc.13.130 PMC4154374

[b6] Daye D. & Wellen K. E. Metabolic reprogramming in cancer: Unraveling the role of glutamine in tumorigenesis. Semin. Cell Dev. Biol. 23, 362–369 (2012). 22349059 10.1016/j.semcdb.2012.02.002

[b7] DeBerardinis R. J. & Cheng T. Q's next: the diverse functions of glutamine in metabolism, cell biology and cancer. Oncogene 29, 313–324 (2010). 19881548 10.1038/onc.2009.358 PMC2809806

[b8] Cairns R. A., Harris I. S. & Mak T. W. Regulation of cancer cell metabolism. Nat. Rev. Cancer 11, 85–95 (2011). 21258394 10.1038/nrc2981

[b9] Kroemer G. & Pouyssegur J. Tumor cell metabolism: cancer's Achilles' heel. Cancer Cell 13, 472–482 (2008). 18538731 10.1016/j.ccr.2008.05.005

[b10] DeBerardinis R. J. *et al.* Beyond aerobic glycolysis: transformed cells can engage in glutamine metabolism that exceeds the requirement for protein and nucleotide synthesis. Proc. Natl Acad. Sci. USA 104, 19345–19350 (2007). 18032601 10.1073/pnas.0709747104 PMC2148292

[b11] Le A. *et al.* Glucose-independent glutamine metabolism via TCA cycling for proliferation and survival in b cells. Cell Metab. 15, 110–121 (2012). 22225880 10.1016/j.cmet.2011.12.009 PMC3345194

[b12] de la Rosa V. *et al.* A novel glutaminase isoform in mammalian tissues. Neurochem. Int. 55, 76–84 (2009). 19428810 10.1016/j.neuint.2009.02.021

[b13] Hu W. *et al.* Glutaminase 2, a novel p53 target gene regulating energy metabolism and antioxidant function. Proc. Natl Acad. Sci. USA 107, 7455–7460 (2010). 20378837 10.1073/pnas.1001006107 PMC2867677

[b14] Cassago A. *et al.* Mitochondrial localization and structure-based phosphate activation mechanism of Glutaminase C with implications for cancer metabolism. Proc. Natl Acad. Sci. USA 109, 1092–1097 (2012). 22228304 10.1073/pnas.1112495109 PMC3268272

[b15] Mohamed A., Deng X., Khuri F. R. & Owonikoko T. K. Altered glutamine metabolism and therapeutic opportunities for lung cancer. Clin. Lung Cancer 15, 7–15 (2014). 24377741 10.1016/j.cllc.2013.09.001 PMC3970234

[b16] Huang F., Zhang Q., Ma H., Lv Q. & Zhang T. Expression of glutaminase is upregulated in colorectal cancer and of clinical significance. Int. J. Clin. Exp. Pathol. 7, 1093–1100 (2014). 24696726 PMC3971313

[b17] Pan T. *et al.* Elevated expression of glutaminase confers glucose utilization via glutaminolysis in prostate cancer. Biochem. Biophys. Res. Commun. 456, 452–458 (2015). 25482439 10.1016/j.bbrc.2014.11.105

[b18] Szeliga M. *et al.* Silencing of GLS and overexpression of GLS2 genes cooperate in decreasing the proliferation and viability of glioblastoma cells. Tumor Biol. 35, 1855–1862 (2014). 10.1007/s13277-013-1247-4 PMC3967065 24096582

[b19] Robinson M. M. *et al.* Novel mechanism of inhibition of rat kidney-type glutaminase by bis-2-(5-phenylacetamido-1,2,4-thiadiazol-2-yl)ethyl sulfide (BPTES). Biochem. J. 406, 407–414 (2007). 17581113 10.1042/BJ20070039 PMC2049044

[b20] Wang J. Bin *et al.* Targeting mitochondrial glutaminase activity inhibits oncogenic transformation. Cancer Cell 18, 207–219 (2010). 20832749 10.1016/j.ccr.2010.08.009 PMC3078749

[b21] Gross M. I. *et al.* Antitumor activity of the glutaminase inhibitor CB-839 in triple-negative breast cancer. Mol. Cancer Ther. 13, 890–901 (2014). 24523301 10.1158/1535-7163.MCT-13-0870

[b22] Wise D. R. *et al.* Myc regulates a transcriptional program that stimulates mitochondrial glutaminolysis and leads to glutamine addiction. Proc. Natl Acad. Sci. USA 105, 18782–18787 (2008). 19033189 10.1073/pnas.0810199105 PMC2596212

[b23] Gao P. *et al.* c-Myc suppression of miR-23a/b enhances mitochondrial glutaminase expression and glutamine metabolism. Nature 458, 762–765 (2009). 19219026 10.1038/nature07823 PMC2729443

[b24] Yuneva M. O. *et al.* The metabolic profile of tumors depends on both the responsible genetic lesion and tissue type. Cell Metab. 15, 157–170 (2012). 22326218 10.1016/j.cmet.2011.12.015 PMC3282107

[b25] Xia Z. *et al.* Dynamic analyses of alternative polyadenylation from RNA-seq reveal a 3′-UTR landscape across seven tumour types. Nat. Commun. 5, 5274 (2014). 25409906 10.1038/ncomms6274 PMC4467577

[b26] Bott A. J. *et al.* Oncogenic Myc induces expression of glutamine synthetase through promoter demethylation. Cell Metab. 22, 1068–1077 (2015). 26603296 10.1016/j.cmet.2015.09.025 PMC4670565

[b27] Qie S., Chu C., Li W., Wang C. & Sang N. ErbB2 activation upregulates glutaminase 1 expression which promotes breast cancer cell proliferation. J. Cell. Biochem. 115, 498–509 (2014). 24122876 10.1002/jcb.24684 PMC4518873

[b28] Kenny J. *et al.* Bacterial expression, purification, and characterization of rat kidney-type mitochondrial glutaminase. Protein Expr. Purif. 31, 140–148 (2003). 12963351 10.1016/s1046-5928(03)00161-x

[b29] Angel P., Hattori K., Smeal T. & Karin M. The jun proto-oncogene is positively autoregulated by its product, Jun/AP-1. Cell 55, 875–885 (1988). 3142689 10.1016/0092-8674(88)90143-2

[b30] Sabapathy K. *et al.* Distinct roles for JNK1 and JNK2 in regulating JNK activity and c-Jun-dependent cell proliferation. Mol. Cell 15, 713–725 (2004). 15350216 10.1016/j.molcel.2004.08.028

[b31] Li Z. *et al.* Regulation of PTEN by Rho small GTPases. Nat. Cell Biol. 7, 399–404 (2005). 15793569 10.1038/ncb1236

[b32] Csibi A. *et al.* The mTORC1/S6K1 pathway regulates glutamine metabolism through the eIF4B-dependent control of c-Myc translation. Curr. Biol. 24, 2274–2280 (2014). 25220053 10.1016/j.cub.2014.08.007 PMC4190129

[b33] Fanjul A. *et al.* A new class of retinoids with selective inhibition of AP-1 inhibits proliferation. Nature 372, 107–111 (1994). 7969403 10.1038/372107a0

[b34] Lei K. *et al.* The Bax subfamily of Bcl2-related proteins is essential for apoptotic signal transduction by c-Jun NH(2)-terminal kinase. Mol. Cell. Biol. 22, 4929–4942 (2002). 12052897 10.1128/MCB.22.13.4929-4942.2002 PMC133923

[b35] Zhao C. *et al.* Genome-wide profiling of AP-1-regulated transcription provides insights into the invasiveness of triple-negative breast cancer. Cancer Res. 74, 3983–3994 (2014). 24830720 10.1158/0008-5472.CAN-13-3396

[b36] Vleugel M. M., Greijer A. E., Bos R., van der Wall E. & van Diest P. J. c-Jun activation is associated with proliferation and angiogenesis in invasive breast cancer. Hum. Pathol. 37, 668–674 (2006). 16733206 10.1016/j.humpath.2006.01.022

[b37] Shao J. *et al.* COP1 and GSK3β cooperate to promote c-Jun degradation and inhibit breast cancer cell tumorigenesis. Neoplasia 15, 1075–1085 (2013). 24027432 10.1593/neo.13966 PMC3769886

[b38] Singh R. *et al.* Differential effects of JNK1 and JNK2 inhibition on murine steatohepatitis and insulin resistance. Hepatology 49, 87–96 (2009). 19053047 10.1002/hep.22578 PMC2614457

[b39] Yang G. *et al.* Isoform-specific palmitoylation of JNK regulates axonal development. Cell Death Differ. 19, 553–561 (2012). 21941371 10.1038/cdd.2011.124 PMC3307970

[b40] Zhao L. *et al.* Interferon-α regulates glutaminase 1 promoter through STAT1 phosphorylation: relevance to HIV-1 associated neurocognitive disorders. PLoS ONE 7, e32995 (2012). 22479354 10.1371/journal.pone.0032995 PMC3316554

[b41] Reynolds M. R. *et al.* Control of glutamine metabolism by the tumor suppressor Rb. Oncogene 33, 556–566 (2014). 23353822 10.1038/onc.2012.635 PMC3918885

[b42] Messeguer X. *et al.* PROMO: detection of known transcription regulatory elements using species-tailored searches. Bioinformatics 18, 333–334 (2002). 11847087 10.1093/bioinformatics/18.2.333

[b43] Koudritsky M. & Domany E. Positional distribution of human transcription factor binding sites. Nucleic Acids Res. 36, 6795–6805 (2008). 18953043 10.1093/nar/gkn752 PMC2588498

[b44] Yang W. *et al.* Genomics of drug sensitivity in cancer (GDSC): a resource for therapeutic biomarker discovery in cancer cells. Nucleic Acids Res. 41, D955–D961 (2013). 23180760 10.1093/nar/gks1111 PMC3531057

[b45] Cerami E. *et al.* The cBio cancer genomics portal: an open platform for exploring multidimensional cancer genomics data. Cancer Discov. 2, 401–404 (2012). 22588877 10.1158/2159-8290.CD-12-0095 PMC3956037

[b46] Gao J. *et al.* Integrative analysis of complex cancer genomics and clinical profiles using the cBioPortal. Sci. Signal. 6, pl1–pl1 (2013). 23550210 10.1126/scisignal.2004088 PMC4160307

[b47] Florin L. *et al.* Identification of novel AP-1 target genes in fibroblasts regulated during cutaneous wound healing. Oncogene 23, 7005–7017 (2004). 15273721 10.1038/sj.onc.1207938

[b48] Eferl R. & Wagner E. F. AP-1: a double-edged sword in tumorigenesis. Nat. Rev. Cancer 3, 859–868 (2003). 14668816 10.1038/nrc1209

[b49] Shaulian E. & Karin M. AP-1 as a regulator of cell life and death. Nat. Cell Biol. 4, E131–E136 (2002). 11988758 10.1038/ncb0502-e131

[b50] Cheng T. *et al.* Pyruvate carboxylase is required for glutamine-independent growth of tumor cells. Proc. Natl Acad. Sci. USA 108, 8674–8679 (2011). 21555572 10.1073/pnas.1016627108 PMC3102381

[b51] Stalnecker C. A. *et al.* Mechanism by which a recently discovered allosteric inhibitor blocks glutamine metabolism in transformed cells. Proc. Natl Acad. Sci. USA 112, 394–399 (2014). 25548170 10.1073/pnas.1414056112 PMC4299208

[b52] Maki Y., Bos T. J., Davis C., Starbuck M. & Vogt P. K. Avian sarcoma virus 17 carries the jun oncogene. Proc. Natl Acad. Sci. USA 84, 2848–2852 (1987). 3033666 10.1073/pnas.84.9.2848 PMC304757

[b53] Bohmann D. *et al.* Human proto-oncogene c-jun encodes a DNA binding protein with structural and functional properties of transcription factor AP-1. Science 238, 1386–1392 (1987). 2825349 10.1126/science.2825349

[b54] Schreiber M. *et al.* Control of cell cycle progression by c-Jun is p53 dependent. Genes Dev. 13, 607–619 (1999). 10072388 10.1101/gad.13.5.607 PMC316508

[b55] Lallemand D., Spyrou G., Yaniv M. & Pfarr C. M. Variations in Jun and Fos protein expression and AP-1 activity in cycling, resting and stimulated fibroblasts. Oncogene 14, 819–830 (1997). 9047389 10.1038/sj.onc.1200901

[b56] Wada T. *et al.* MKK7 couples stress signalling to G2/M cell-cycle progression and cellular senescence. Nat. Cell Biol. 6, 215–226 (2004). 15039780 10.1038/ncb1098

[b57] Shaulian E. & Karin M. AP-1 in cell proliferation and survival. Oncogene 20, 2390–2400 (2001). 11402335 10.1038/sj.onc.1204383

[b58] Goodrich D. W., Wang N. P., Qian Y. W., Lee E. Y. & Lee W. H. The retinoblastoma gene product regulates progression through the G1 phase of the cell cycle. Cell 67, 293–302 (1991). 1655277 10.1016/0092-8674(91)90181-w

[b59] Mariani O. *et al.* JUN oncogene amplification and overexpression block adipocytic differentiation in highly aggressive sarcomas. Cancer Cell 11, 361–374 (2007). 17418412 10.1016/j.ccr.2007.02.007

[b60] Beroukhim R. *et al.* The landscape of somatic copy-number alteration across human cancers. Nature 463, 899–905 (2010). 20164920 10.1038/nature08822 PMC2826709

[b61] Kappelmann M., Kuphal S., Meister G., Vardimon L. & Bosserhoff A.-K. MicroRNA miR-125b controls melanoma progression by direct regulation of c-Jun protein expression. Oncogene 32, 2984–2991 (2013). 22797068 10.1038/onc.2012.307

[b62] Bakiri L., Matsuo K., Wisniewska M., Wagner E. F. & Yaniv M. Promoter specificity and biological activity of tethered AP-1 dimers. Mol. Cell. Biol. 22, 4952–4964 (2002). 12052899 10.1128/MCB.22.13.4952-4964.2002 PMC133900

[b63] Mechta-Grigoriou F., Gerald D. & Yaniv M. The mammalian Jun proteins: redundancy and specificity. Oncogene 20, 2378–2389 (2001). 11402334 10.1038/sj.onc.1204381

[b64] Smith L. M. *et al.* cJun overexpression in MCF-7 breast cancer cells produces a tumorigenic, invasive and hormone resistant phenotype. Oncogene 18, 6063–6070 (1999). 10557095 10.1038/sj.onc.1202989

[b65] Liu Y. *et al.* AP-1 blockade in breast cancer cells causes cell cycle arrest by suppressing G1 cyclin expression and reducing cyclin-dependent kinase activity. Oncogene 23, 8238–8246 (2004). 15378019 10.1038/sj.onc.1207889

[b66] Wang X. *et al.* Elevated expression of phosphorylated c-Jun NH2-terminal kinase in basal-like and ‘triple-negative' breast cancers. Hum. Pathol. 41, 401–406 (2010). 19913278 10.1016/j.humpath.2009.08.018

[b67] Horiuchi D. *et al.* MYC pathway activation in triple-negative breast cancer is synthetic lethal with CDK inhibition. J. Exp. Med. 209, 679–696 (2012). 22430491 10.1084/jem.20111512 PMC3328367

[b68] Wang Z.-Y. *et al.* Regulation of IL-10 gene expression in Th2 cells by Jun proteins. J. Immunol. 174, 2098–2105 (2005). 15699140 10.4049/jimmunol.174.4.2098

