# Supplementary material for: The oncogenic transcription factor c-Jun regulates glutaminase expression and sensitizes cells to glutaminase-targeted therapy
Source: Nat Commun. 2016 Apr 18;7:11321. doi: 10.1038/ncomms11321 (PMC4837472; doi:10.1038/ncomms11321)
Supplement: Supplementary Information — Supplementary Figures 1-16 and Supplementary Tables 1-2 [file ncomms11321-s1.pdf]

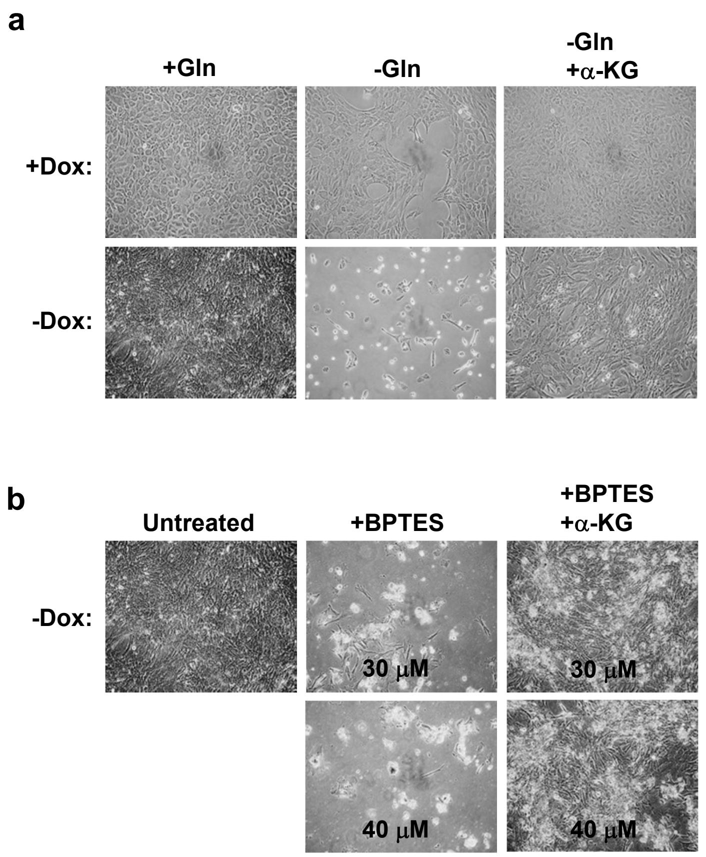

Supplementary Figure 1. Glutamine-dependent transformation by oncogenic-Dbl. Phase-contrast microscopy (100× magnification) of the samples shown in Fig. 1d and 1g, taken prior to fixation and staining. Panel (a) corresponds to Fig. 1d, and panel (b) corresponds to Fig. 1g.

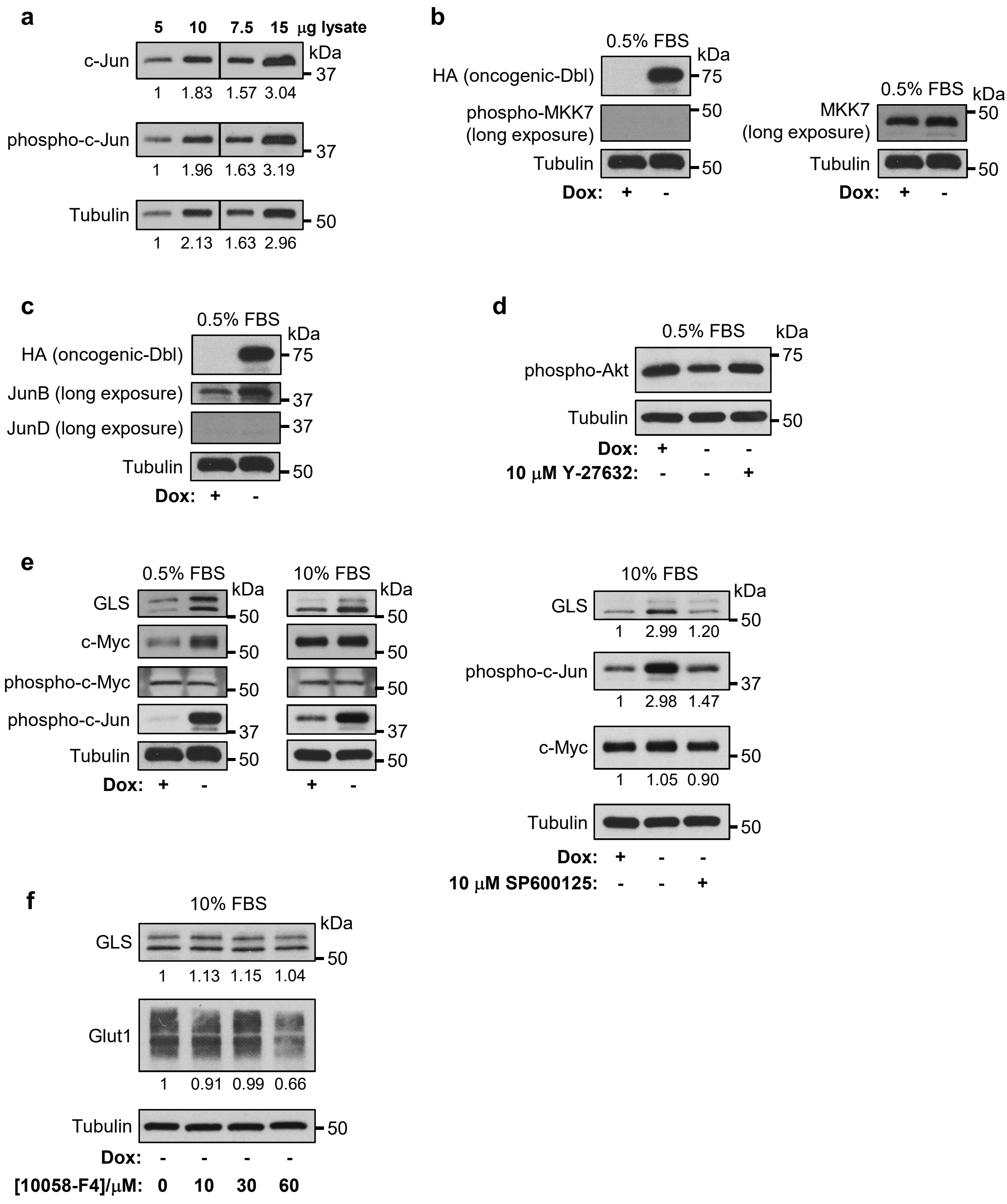

Supplementary Figure 2. Signaling processes downstream of oncogenic-Dbl. (a) Western blot analysis confirming signal linearity for the c-Jun and phospho-c-Jun antibodies. 5-15 μg of whole-cell lysate from induced MEFs was loaded onto a single gel. (b) Western blot analysis of whole-cell lysates of MEFs in which oncogenic-Dbl

expression was either uninduced (+Dox) or induced for 24 h (-Dox) under low-serum (0.5% FBS) conditions. In contrast to MKK4, which is potently activated upon oncogenic-Dbl induction (Fig. 2a), activated MKK7 could not be detected by western blot. Total MKK7 was detectable, although long exposure times were required. (c) In contrast to c-Jun, which is potently activated downstream of oncogenic-Dbl (Fig. 2a), phospho-JunB could not be detected by western blot (not shown). Total JunB was elevated in induced, relative to uninduced, MEFs, but very long exposures were required to detect this signal. Total JunD was barely detectable. The same antibodies yielded strong signals with exposure times shorter than 10 seconds in some breast cancer cell lines (see Fig. 4b) and are predicted to recognize the mouse and human proteins equally well, indicating that JunB and JunD are not abundant in the MEFs. (d) Western blot analysis showing changes in Akt phosphorylation downstream of oncogenic-Dbl signaling. Induction of oncogenic-Dbl expression leads to a moderate decrease in Akt phosphorylation at residue T308. Treatment of induced MEFs with the ROCK inhibitor Y-27632 (10 μM) largely reverses this decrease, consistent with previous reports that activation of ROCK by Rho GTPases leads to down-regulated Akt phosphorylation. (e) Left panels show changes in c-Myc, GLS, and phospho-c-Jun levels downstream of oncogenic-Dbl under different serum concentrations. In low-serum (0.5% FBS) media, induction of oncogenic-Dbl leads to elevated GLS, c-Myc, and phospho-c-Jun levels. c-Myc phosphorylation (residues T58/S62) is decreased, possibly reflecting decreased targeting for proteasomal degradation. Under 10% FBS, induction of oncogenic-Dbl does not affect the level of c-Myc (which is high in both uninduced and induced cells) or phospho-c-Myc, but still leads to increased c-Jun phosphorylation and elevated GLS. Right panel shows that in high-serum (10% FBS) media, phosphorylation of c-Jun and elevation of GLS downstream of oncogenic-Dbl are inhibited by the JNK inhibitor SP600125 (10 μM), as is also the case under 0.5% FBS (see Fig. 3a). (f) GLS levels in induced MEFs under different concentrations of the c-Myc inhibitor 10058-F4 (48 h treatment). Concentrations up to 60 μM had no effect on GLS levels, whereas expression of the c-Myc transcriptional target Glut1 was suppressed.

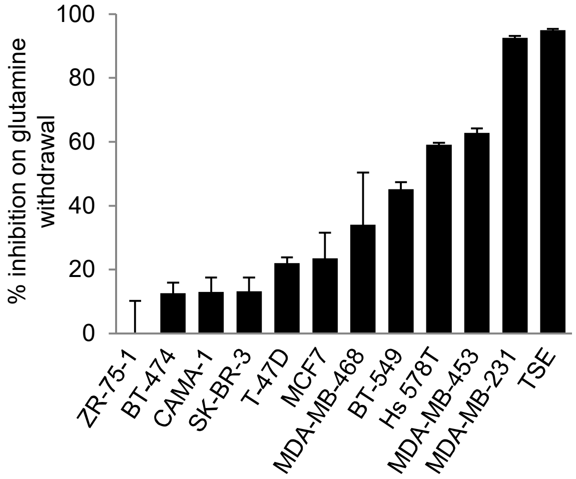

Supplementary Figure 3. Glutamine dependence of breast cancer cell lines. Proliferation assays were carried out for the indicated cell lines in complete RPMI medium containing 2.0 mM or 0.1 mM glutamine, and supplemented with 10% FBS. The inhibitory effect of glutamine withdrawal was calculated as a percentage. The data presented are the mean ± SD of triplicate proliferation assays.

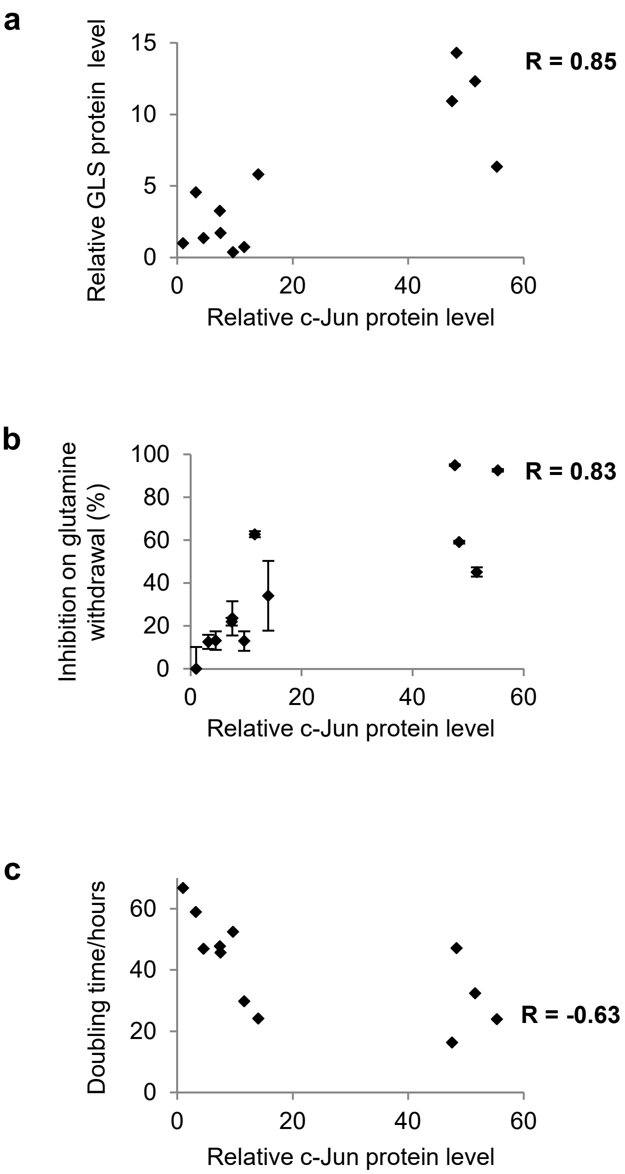

Supplementary Figure 4. c-Jun levels correlate with GLS levels, glutamine dependence, and proliferation rate across 12 human breast cancer cell lines. (a) Quantification of western blot band intensities for GLS and c-Jun from Fig. 4a, showing a positive correlation with a Pearson coefficient of 0.85. (b) Relative c-Jun protein levels determined from Fig. 4a plotted against glutamine dependence of the cell line, as determined from the inhibition (%) of proliferation over 6 days that occurs when glutamine levels are lowered from 2 mM to 0.1 mM. A strong positive correlation with a Pearson coefficient of 0.83 is revealed. (c) Relative c-Jun protein levels determined from Fig. 4a plotted against doubling time. Cell lines with shorter doubling times (i.e. more rapidly proliferating) tend to have higher c-Jun levels. The Pearson correlation coefficient is -0.63.

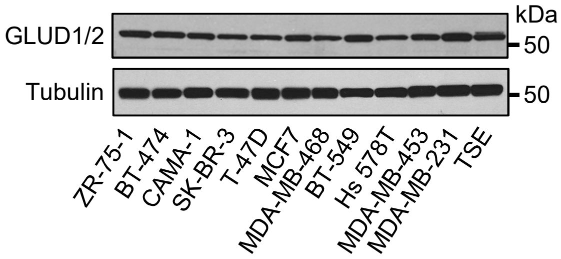

Supplementary Figure 5. Western blot analysis showing relative GLUD1/2 levels in breast cancer cell lines. Cells were harvested at ~60% confluency from RPMI growth medium supplemented with 10% FBS. Samples were ordered according to relative glutamine dependence (increasing left to right).

```
GGCTACCACGTCTTCTTCATTCAAATTGGACGATATATCAGTCAGCTTAAGCTAAGTCATTCTATGGTAATAACCCTCAAGTTTAAATGACTTAAAATAAGGCTTATTTCTCTT
GATGTTATTTGTCAGCCAAAGCCATTGTCAGCTTTGTTCCATAATTCTGGGACCCAGGTAAAGAAGCATCCCCTCATGGACTGGACTAGAACCAATGAACTGTGGTGGGGTGTG
GGGGGAAAGAAGCGTCCCCTCTCTGGGACATGCCATTCTTGTTTAAAAGGGAAAAGAGAGATGACAGAACCAAGTGAGTTTATGCTCGGCAGTGGCATACCACTTCCTGTCACA
TATCATTGAACAAATCAAGGCCAAGACTGCCATCCAAGGTGAGGAGGTAAAGTCTGCCCAGTGGGAGGGACAAAAATATTTGGGAATAGTAGTATAATCTACCACAGAGGTTGT
TTTCATTCTCAAGCAGGTATCCTTTTGACTGACTTTTGGACTATATACAAAATCAGTTTTTTTTAGGTTTTGCTAATATACAAGAAAGACTTTCTTAACTCTGGGCTGGGGTTT
AGCCTGATTTATTTTTAATATGAATCCACAGAAAAATAAGCATTAAAATCACTTTAAAAAATTAATATTTGGAGGCCCCATAAATATAATGGTGGTGTAAATATAAACAATATT
ACCTTACAAACTCCACTAATTGAAATCTTTATCATATTACTCTAAGTGGAGAAAAATAACTGATTCCCACCCAACCCCCACTCCGGCCATCAGGAAACTCTCAAAAAGAACTTC
ATATAGTTAGTATTTTAAGGCAGATCATTCAGATATGCTGCTCAAATTTGATGAATATCTCTTGTCATTTTATATGATGTCACAATGAACAGAGGTATACTTTCATTTACATAG
ATGGCAAATGATATATTTTTAGATTTAATTGCTATATGTTAAGGTATTTGTATATGACATGTATATTCAAGGACTGCTTTCTTAGCGATTCTTCCTTCTCTGGAATTTTTTTTT
TTTTTTAGCTATTTGTGAATAGCTGTTACTTTGGGCACTGAGCCCAGCTTTTCTTAATCTGACTGTCTTTATCTTTTTCTATTTTAAGATACTTTGAAAATTATTGGTTTATTT
TCTAAAAATGAAGTAAGTAAATTATCTCTAGGTTCCCCTTAAATGGTATTTTATTATTAGATGGTTTAAAACAATTGTTTGGCTTTTCAACAATAAGTGTAAAAACTGATCACA
TTGCATGTCAAAATTTATTCACCTATGGTTTGATAATAACAGCTATAACACTTACAGAGTGCTACTGTCATTATTCTGGGTGCTTTATATGTGTTGTTTATTTATTCCACACAA
CAACCCTATAAGTACCAATATTATCCGCCCCCCTTTGTTTTTTTGAGATGGAGTCTTGCTCTCTCATAGCCCAGGCTGGAGTGCAATGGCTAGATCTCGGCTCACTGCAACCTC
TGCCTCCCAGGTTCAAGCAATTCTCCTGCCTCAGCCTCCCGAGTAGCTGGGATTACAGGCATGCACCACCATGCCTGGCTAATTTTTTGAATTTCTAGTAGAGATGGGGTTTCA
CCATGTTGGCCAGGCTGGTCTTGAACTCCTGACCTCAGGTGATCCACCTGCTTCGGCCTCCCAAAGTGCTGGGATTACAGGCGTGAGCCACCTTGCCTGGCCATATTATCCCCT
TTTTATAGATGAGGGATTTACCATACTTACTTTGGTACACATCTATATTCAGTATTGGAAAAATAAGCCATGTTTGGCAGGGCTTATCAGTGAGGCTAGGAATTTGGCCAGTTA
AAGCAGGAAACCAACAGAAGAAAGGGAAGTAGTAGCAGAAGCTTGTTTAGAGGCTTCCTGAACCTTACTCTATTCTCCAAACAGAAGAATTTCAAAAAAAGGCTTAGGCAATTC
TTTTAAGCTGTTAGTCAAAGGAAAATTACTTTCTTTTTAGTTCTAGGTCAACAAATAATCTACTAGTTTTTGTGAAGGCGCAATCTGGAATTTCTATATATATATATTATTTTA
TCTTATTTTATTTTATTTTTTGAGATGTAGTATCGCTCTGTTGCCAGGCTGGAGTGCAGTGGTGCGATCTTGGCTCACTGTAACCTCGGCCTCCCAGGTTTGAGTGATTCTCCT
GCCTCAGCCTCCCAAGTAGCTGGGATTACAGGTGTGTGCCACCACGCCCAGCTAATCGTTGTATTTTTAGTAGAGATGGGGTTTAACCGTGTTAGCCAGGATGGTCTCAATCTC
CTGACCTTGTGATCTGCTCGCCTCAGCCTCCCAAAGTGCTGGGATTACAGGTGTGAGTCCACCGCGCCCGGCCTATATATGGATTTTATTATGATCTTGAAACATTAGCCTTTT
GTAATCTTCGACAGAACAAAGAAGCTCAGGAACATAGAAGCACAGGAACCTTTCTTCAAGTTAGAACAGACACAAAGTGCACAAATTCCAATTATTCCTTTTGATGGCTGATTA
CCTGATGATGGCTATGGGACAGCAACTCTTTGTAGTGTAGCTGCACTTAGGAGCAAAAAGGAAGTCGAAGAGTAGATCTGACAACCCAACCATAGTTAATGTGGAGAGCATTTT
TCTATTTGTAGATATTTTGGTGGACTACTTATTGTATGTATACAAATGTTTTTATACTTTTTACATGATGATTACATGAGTTTATGTAATGATATAATTCAATTTAACATAACT
GAAACTTCAAAGTTGTGTAATGGTTACAAAGTTGTAGATAAAAATATCTTTAAAGATTAAAAAATTATATAAGATGAAGAAAAGAATCAAATGAAAGTATTGAGTCACATTTTT
ATTCTATTTATAATAGTTTCTTTGCTGTCTATAACCTTTTCATTAGCCTTCTCAAACAAGGGGTTAAATTTCAGTGGACTCAATTCTTCCAAATTTTGGGAGTAATATCTATCC
ATTCTCACCAAAAGCAGAAAGTCTGGAAACTACCAGAAAATAAAACTCTAGAAACTAGAAACTCTAGAAAATAATCTGTTACTAGGTTAGCTTTCTTATTTGTTAAGTGAACTA
TAAAGCCTTTAAACCTTTAAATATCTAAAACAATTTTCATAAGAAACTCAGAGAAATTAAGGGAGAAACTGAGAGGAAAACAGTAACTGGATTGTCAGTTGCTTTGTGTTGTTG
AGAAAAAAGAGGCAGTTAAAATTGATAACTAATTGCAGCTAATATATTGGACAATATTAAAGTCTGCAAGTCATTAACAAGTCAGGGTTAGGCAGGGGTAATATTTGTTACCTT
TCCTCTGAAACTTGATGTCTCTATTCCTGAATATATCATTACTTTGATTCATCAACATTTTTGGACAAGTAACATTAGTAATAAAAGCTATCTAAGTACTTTCCCATTGTCCTG
CTTTTTTTTTTTTAAATAAAAAAGGTGACTATGTTTTACACATGATAGTTTTTTCCATGTTCTTCTGGTTAAAGAGCAGATAGAAAAAAAAAACTTAAAACACGGCAAGGCTGT
AACATGCCAAAGGTCACATAACCTGTAGGTACAGCCAGATTTCCTAATTATTAATCTATTGAAATATTTTACAGTTCACTAGATTACTAAATCCGGTTATCAATTAATGATGCA
CCTGACATACACCCTTCTAGTTTCATAAAAATCACCTCATTAGGCCGGGCGTGGTGGCTCACGCCTGTAATCCCAGCAATTTGGGAGGCCCAGGGGGTGCAGATCCCTCGAAAC
CAGCCTGGGCAACCTGGCGAAACCCCGTCTCTACCAAAAATACAAAAAAAAAAAAAAAAAAAAAGCAGGTGCTGGCTGAGGTGGGAGGATCACCGGGGCCCCTGAGTTCAAGGCT
GCAGTAAGCCGTGATCGCGCCACTGCACTTCAGCCTGGGCGACAGAGTGAGACCCTTTCTCAAAACGAACACACACAACAACACAGTAACAACACACCAACCCCCAAAACATCT
TATTAAACTCTGTGGATCTACTCCATTTAAACCTAATTGTTTCAGGAAATAGTCTAAAAACATTTTTTTGTCCTGGAATGTAATGTGTATGTAGCCTCAGGGAATAACTCAGTT
TATCAGGTTCTTGGCTGTTTTTCCACAGGTCCAAAAGGATAAATGGATAAAGTGATGCTGAAATAACTTAGAGAACCCGTATTCCAACGCTTCTATTTTAAAACAGTGAAGCAG
TTTTTAAAATAGAAAAGTATCTACAGTTTTGCTTTCAAGCAAATAGCGTTAACGGAATCATTGCTATTCAAAACTATTTTTGAAGTATCGGTGTACATTTAAAAAAAATAATTT
TTAAAAAAGAAAGCTGTCGTATTCTGGATTCCGCAACCCCCCCACCCCGCCTCGCCACTCGTCGCCCTCCCTCCACCTCCACCCTCCCATGGCTTAAGTCGCATAGCTGAAAGG
ATTTTGCTCATTTTTTCCAAAACACTTCACGTCTGGGTAGTAGCCAATCATGGAAAGTTTCAAGCAAATGTTACATTTGGGAGAGGGATGAAGCTTGTCCGGGGCTGCTCTCGG
AGGAGTCGCCAGACGCCCTGACCTGGGGGCCAGATGCGAGCTGGGATCGGCTGAGTTCCGACAGCTCGGCCAGACTCAGCCCTGCCAAACCTGCTGACCTCACTGCCCTCGGCT
GCGGGGGATCTCGCCCATGAGTCTCCCCAACAGCTCGAATTCCCAGAGTGGAGGAGCCCACTGCTTCATAAATGCGAGAACCCAGAGCCCCTAGTACCCAACTAGGCTAGCCTC
GGAGTTGGCACGGCGTGCAGAAAGTGGCTACTGAGCAGCCGGGCCTGGCTCACCCGCTTCACACGTCAGTTTGACTCCTCTCCCCGCCCCCCACCAATCCCCCGCCCTACTCTC
CGCGCACCCAGAGCCGAGAGAAATTTGACTGCCCTAATCGCCAATCAAAAGAGAGGTCGAGGATTTGAGCCAATCGCAGCGGGCGAAAGGGGCGGGCCGTCGGCGAGCGCTGAA
GGTCTCTAGCCCTCCCCTGCGCTTTAGCCTCAGTGCGGAGCCTTAGGCGGAGCGAAGAGAACCGGTCGCGGCAATCCTAGCGCGCAGCAGCAGCAGCAGCAGCAGCAGCAGCAG
CAGCAGCAGCAGCAGCACCCGCATCCGCTGCGGGAGTCCGAGCCGGAACCACACCCAAGTAGCTGCCCTTTCCTCTTCTGTCATCTCACCGCCCCACCACAGACCGCGTTCCCC
GAGGAAACCGGCCGCCCACGCCCGGAGCATCCTCCCCTGTTGAGCGGGCGCTGACGGACCCGGCGGCATG
```

Supplementary Figure 6. Predicted c-Jun binding sites in the human *GLS* promoter. The promoter region for the human *GLS* gene is shown, from 5,046 bp upstream of the transcription start site (TSS) to 264 bp downstream of the TSS. Potential binding sites for c-Jun (dissimilarity from the consensus sequence <15%, determined using the PROMO[42] resource) are highlighted in yellow, the TSS is highlighted in pale green, the 5'-UTR is highlighted in grey, and the START codon highlighted in dark green. A near-perfect match to the c-Jun consensus motif (calculated 2.65% dissimilarity) at position -188 bp relative to the TSS is marked with a box.

a

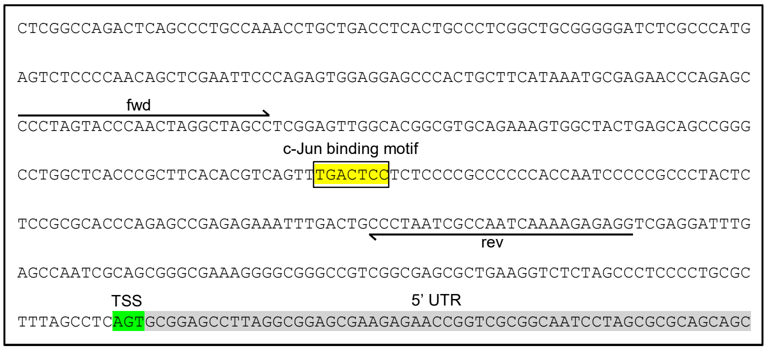

b

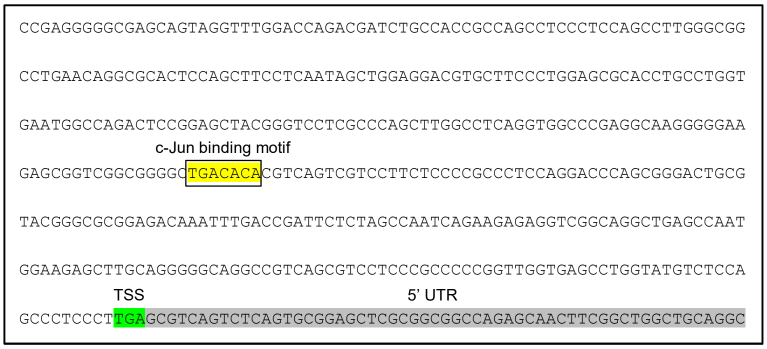

Supplementary Figure 7. The proximal region of the *GLS* promoter. (a) Proximal region of the human *GLS* promoter, showing the transcription start site (TSS) and the predicted c-Jun binding motif at position -188 bp relative to the TSS. The binding sites for forward and reverse primers, designed to amplify a 196 bp fragment centered on the putative c-Jun binding site, are also indicated. These primers were used for the ChIP experiment shown in Fig. 5a. (b) Proximal region of the mouse *GLS* promoter, showing the TSS and the predicted c-Jun binding motif at position -200 bp relative to the TSS.

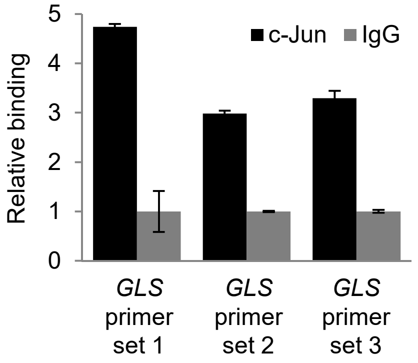

Supplementary Figure 8. Chromatin immunoprecipitation (ChIP) analysis showing that c-Jun binds to the proximal region of the *GLS* promoter. Complexes containing c-Jun were immunoprecipitated from cross-linked, digested chromatin isolated from MDA-MB-231 breast cancer cells. A parallel immuno-precipitation using rabbit IgG was carried out as a negative control. Following reversal of cross-links and purification of DNA, RT-PCR was carried out using three sets of primers designed to amplify a region of the *GLS* promoter centered on a putative c-Jun binding site at position -188 bp relative to the transcription start site. The data presented are the RQ values, with error bars marking RQ max and RQ min, from triplicate reactions.

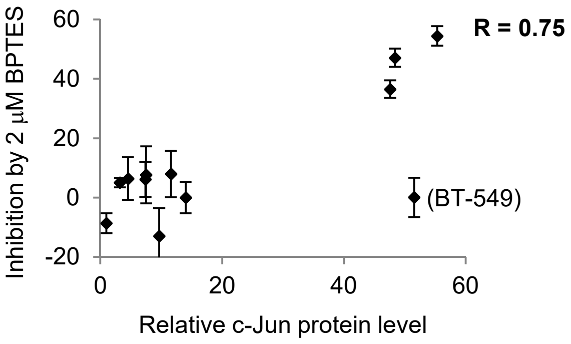

Supplementary Figure 9. c-Jun levels and BPTES sensitivity of breast cancer cell lines. Relative c-Jun protein levels determined from Fig. 4a plotted against sensitivity of the cell line to GLS inhibition, as indicated by inhibition of proliferation over 6 days by treatment with 2 μM BPTES. A strong positive correlation with a Pearson coefficient of 0.75 is seen. The drug-resistant cell line BT-549 is marked, and is a clear outlier

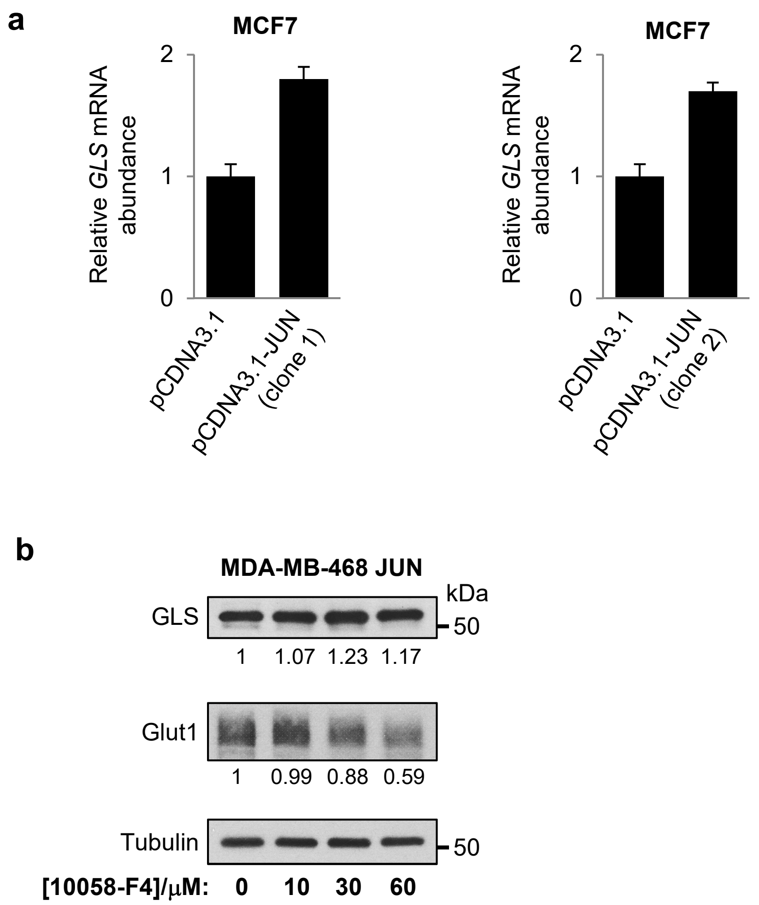

Supplementary Figure 10. Over-expression of the *JUN* oncogene drives *GLS* expression in breast cancer cell lines MCF7 and MDA-MB-468. (a) RT-PCR analysis showing relative *GLS* mRNA abundance in derivative MCF7 cell lines stably carrying either pCDNA3.1 or the *JUN* expression vector pCDNA3.1-JUN. The data presented are the RQ values, with error bars marking RQ max and RQ min, from triplicate reactions. (b) Control blot for Fig. 6e showing that the c-Myc inhibitor 10058-F4 suppresses expression of the c-Myc transcriptional target Glut1, but does not suppress levels of GLS in *JUN*-over-expressing MDA-MB-468 cells.

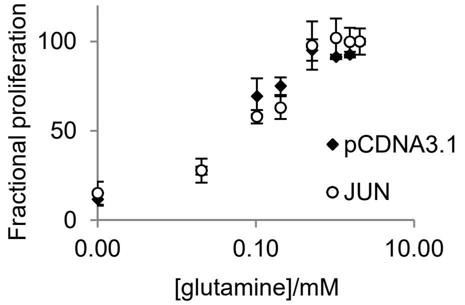

Supplementary Figure 11. Both vector control and *JUN*-over-expressing MDA-MB-468 cells are dependent on glutamine for proliferation. Proliferation assays were carried under a range of glutamine concentrations. Both vector control and *JUN*-over-expressing MDA-MB-468 cells are dependent on glutamine, with no significant differences in the inhibition of proliferation upon glutamine withdrawal.

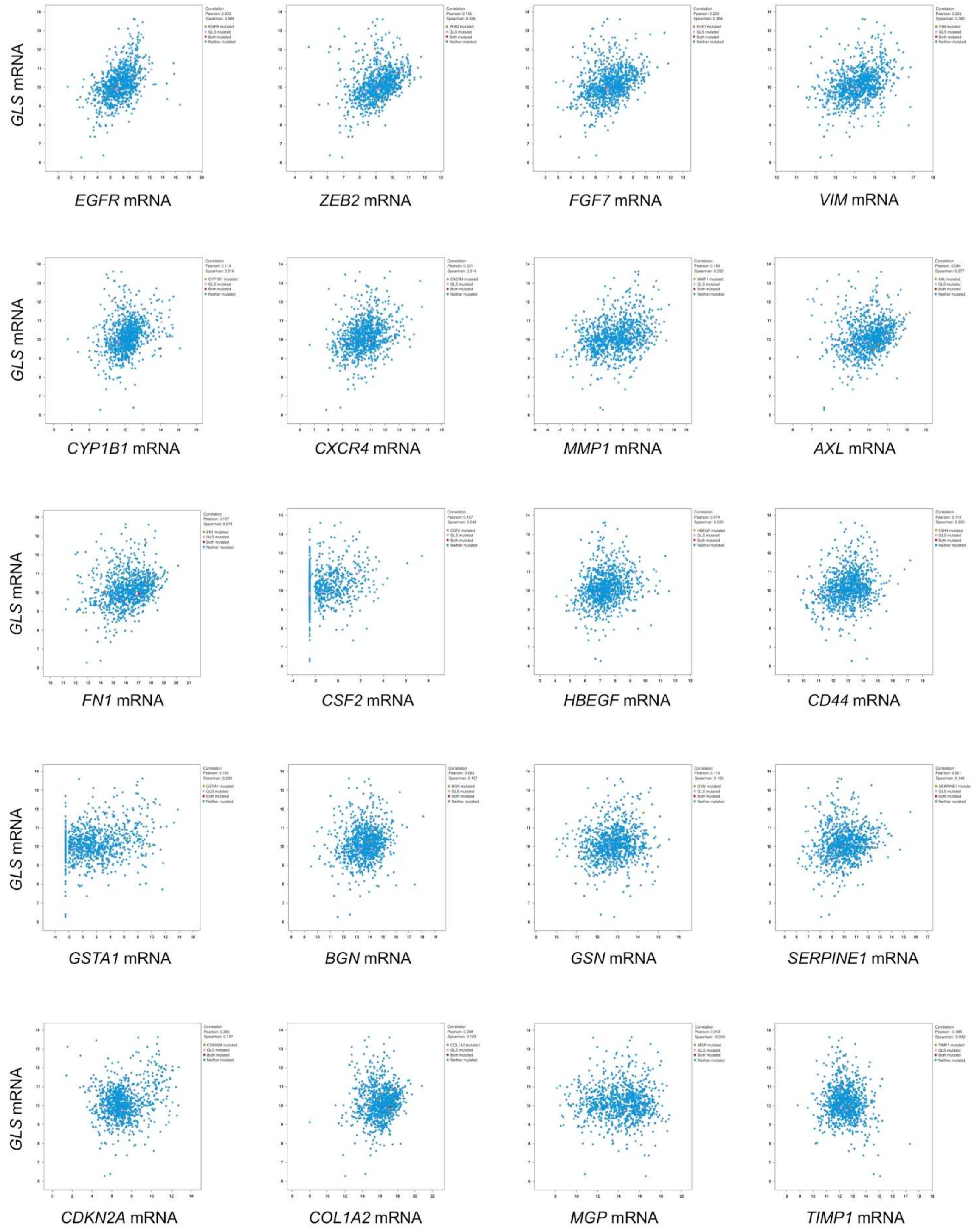

Supplementary Figure 12. Correlation plots for the *GLS* transcript and positively-regulated c-Jun target transcripts in invasive breast cancer. Scatter plots show *GLS* mRNA levels against mRNA levels of established, positively-regulated, c-Jun transcriptional targets. Spearman correlation coefficients are tabulated in Supplementary Table 1. Data are from The Cancer Genome Atlas (TCGA) Breast Invasive Carcinoma (TCGA, provisional) dataset. Plots were prepared, and correlation coefficients determined, using the cBioportal suite of tools.

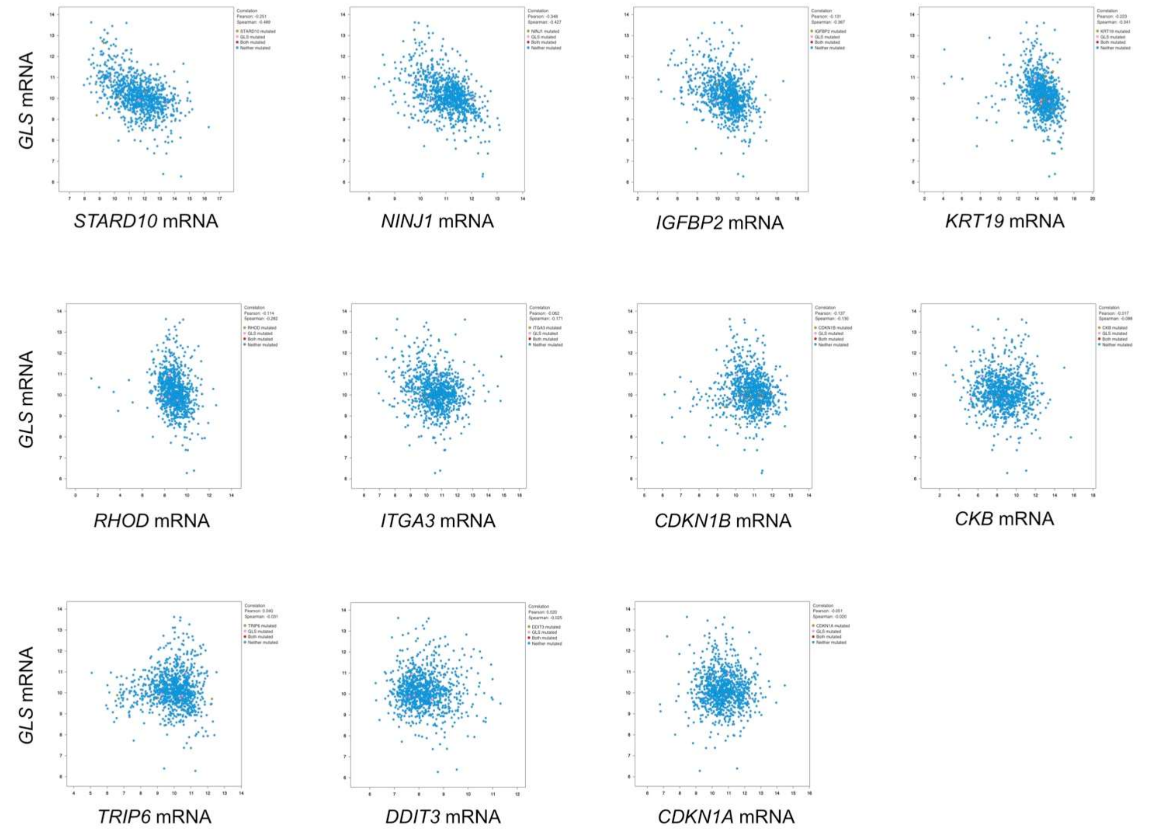

Supplementary Figure 13. Correlation plots for the *GLS* transcript and negatively-regulated c-Jun target transcripts in invasive breast cancer. Scatter plots show *GLS* mRNA levels against mRNA levels of established, negatively-regulated, c-Jun transcriptional targets. Spearman correlation coefficients are tabulated in Supplementary Table 1. Data are from The Cancer Genome Atlas (TCGA) Breast Invasive Carcinoma (TCGA, provisional) dataset. Plots were prepared, and correlation coefficients determined, using the cBioportal suite of tools.

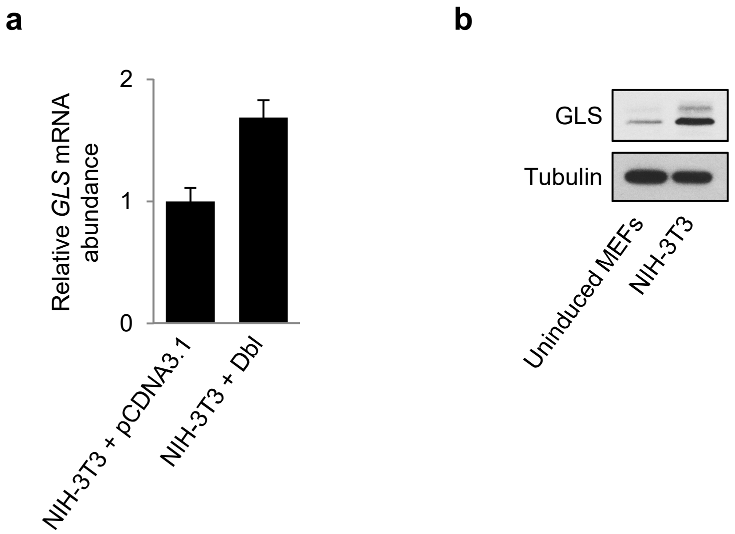

Supplementary Figure 14. Analysis of *GLS* expression in NIH-3T3 cells. (a) RT-PCR analysis showing that transient expression of oncogenic-Dbl in NIH-3T3 cells leads to increased levels of *GLS* transcript. The data presented are the RQ values, with error bars marking RQ max and RQ min, from triplicate reactions. (b) Western blot analysis comparing GLS protein levels in NIH-3T3 cells with those in the inducible MEF cells (uninduced) used in this study. When both cell lines are grown under 10% serum, GLS levels are much higher in NIH-3T3 cells.

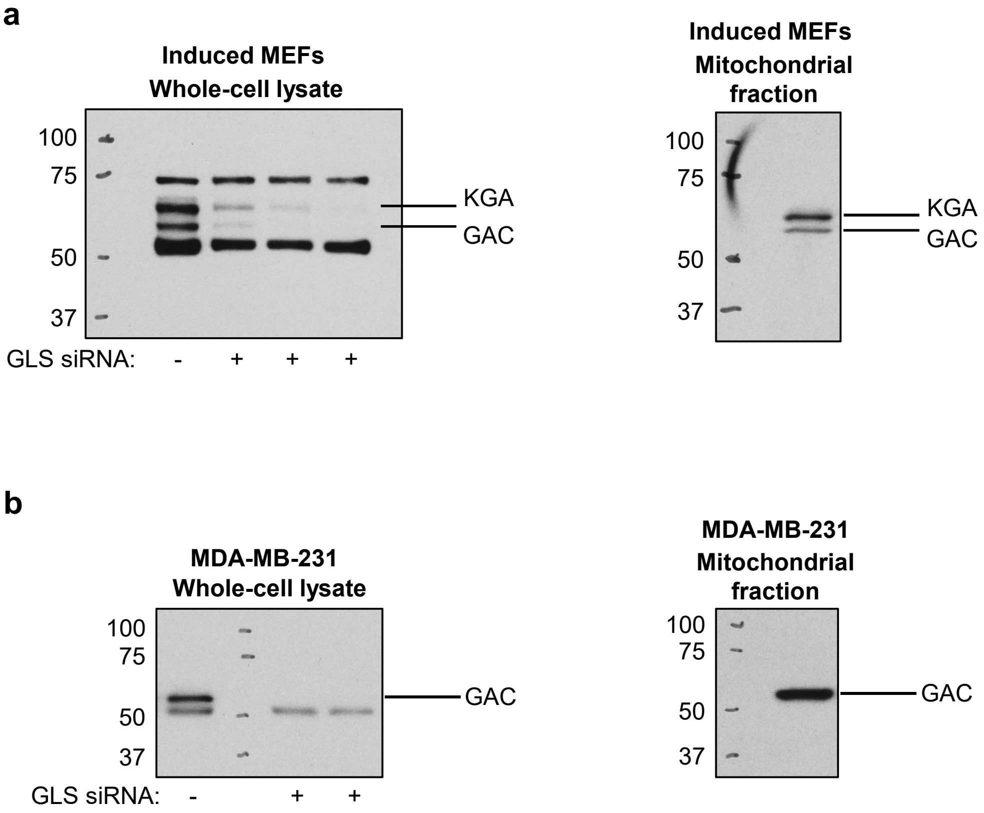

Supplementary Figure 15. Clarification of bands recognized by GLS antibody. (a) In the inducible MEF cell line, the GLS antibody (Abgent, AP8809b) recognized four bands. Two of these bands ran at the molecular weights for the two GLS isoforms, GAC (58 kDa) and KGA (65 kDa). These bands were depleted in cells treated with siRNA targeting GLS (both isoforms), whereas the other two bands were relatively unaffected. The bands corresponding to GAC and KGA were present in mitochondrial fractions, whereas the other bands were not. Since the other bands do not correspond to the molecular weight of known GLS isoforms, are not depleted by GLS-targeted siRNA, and are not mitochondrial, we conclude that they are non-specific. We note that in both MEFs and breast cancer cells (see below), the ~52 kDa non-specific band tends to be more abundant under low serum conditions and when cells are at higher confluency. (b) In most of the human breast cancer cell lines studied, including MDA-MB-231, we could only detect a GLS band corresponding to the molecular weight for GAC, consistent with reports that this GLS isoform is selectively up-regulated in diverse cancer types. A band at the molecular weight for KGA was also detected in BT-474 cells (Fig. 4a). In all cancer cell lines, an apparently non-specific band close to 52 kDa was detected, as in MEFs. This band was not strongly affected by GLS-targeted siRNAs, and was not present in the mitochondrial fraction.

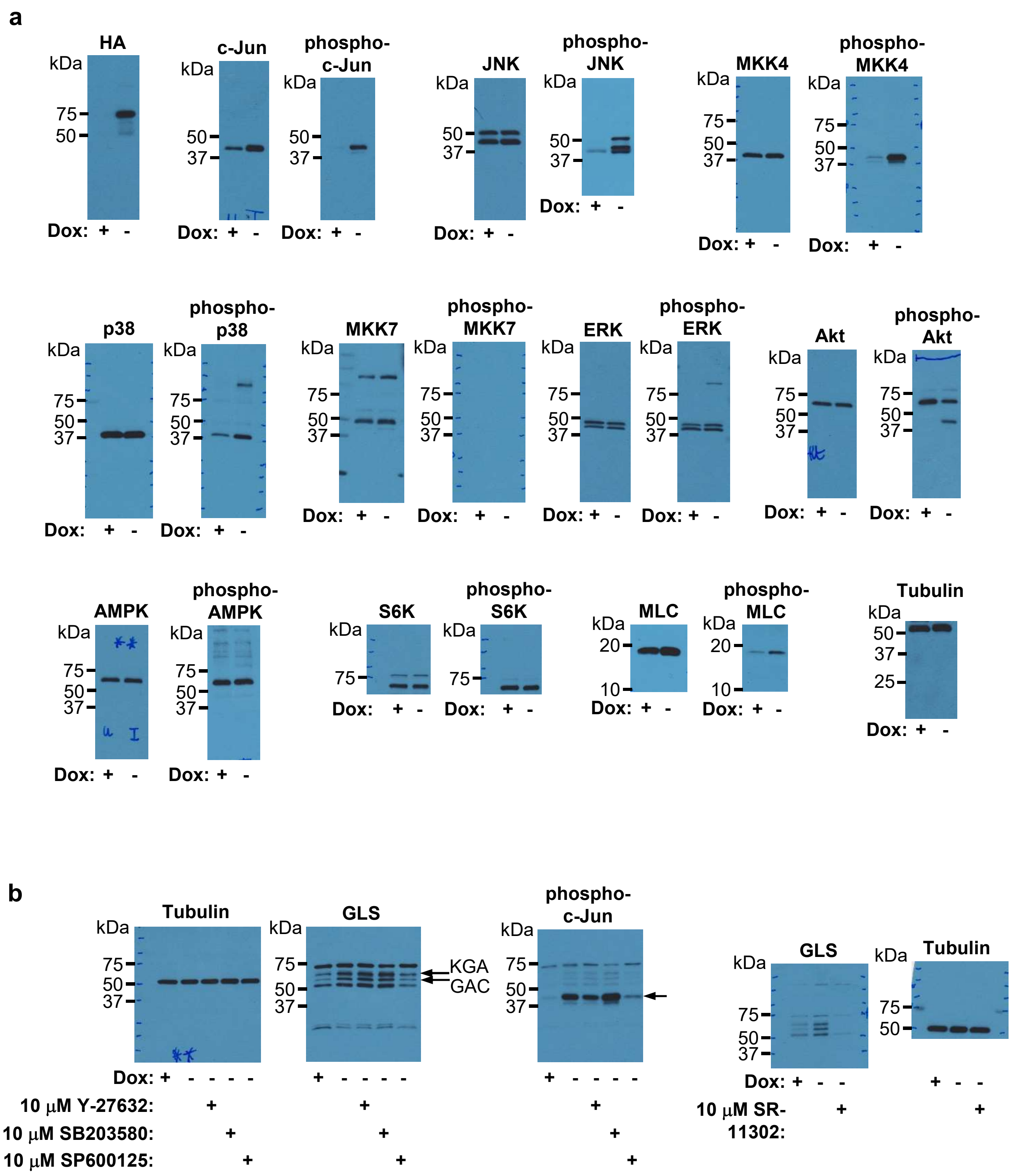

Supplementary Figure 16. Uncropped western blot scans. (a) Inducible oncogenic-Dbl MEFs, signaling pathway experiments. (b) Inducible oncogenic-Dbl MEFs, inhibitor treatment experiments.

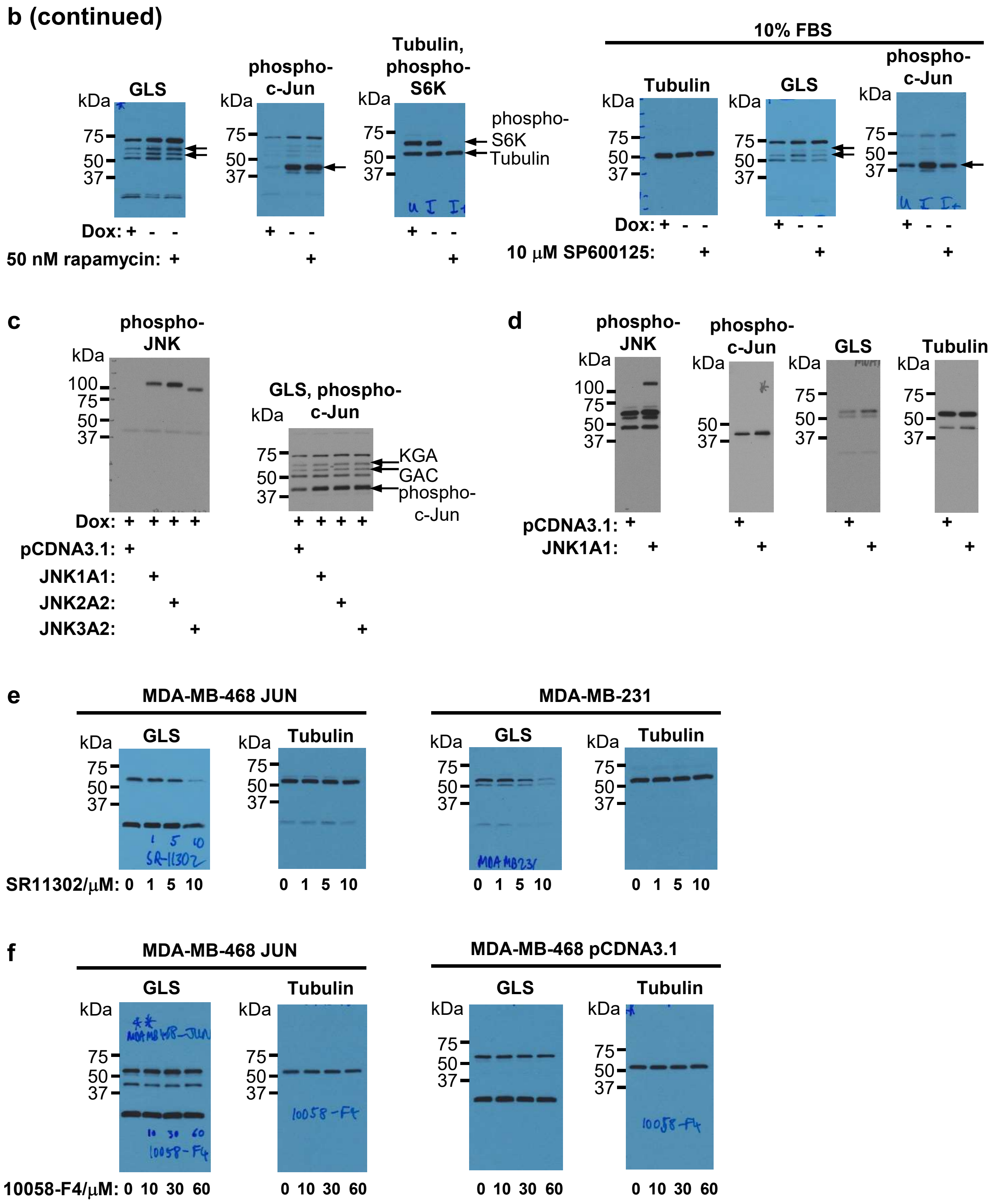

Supplementary Figure 16 (continued). (c) Transient ectopic expression of constitutively activated JNK constructs in uninduced MEFs. (d) Transient ectopic expression of constitutively activated JNK1A1 in MDA-MB-231 cells. (e) SR11302 treatment of MDA-MB-468 (JUN stable derivative) and MDA-MB-231 cells. (f) 10058-F4 treatment of MDA-MB-468 cells (JUN stable derivative and vector control).

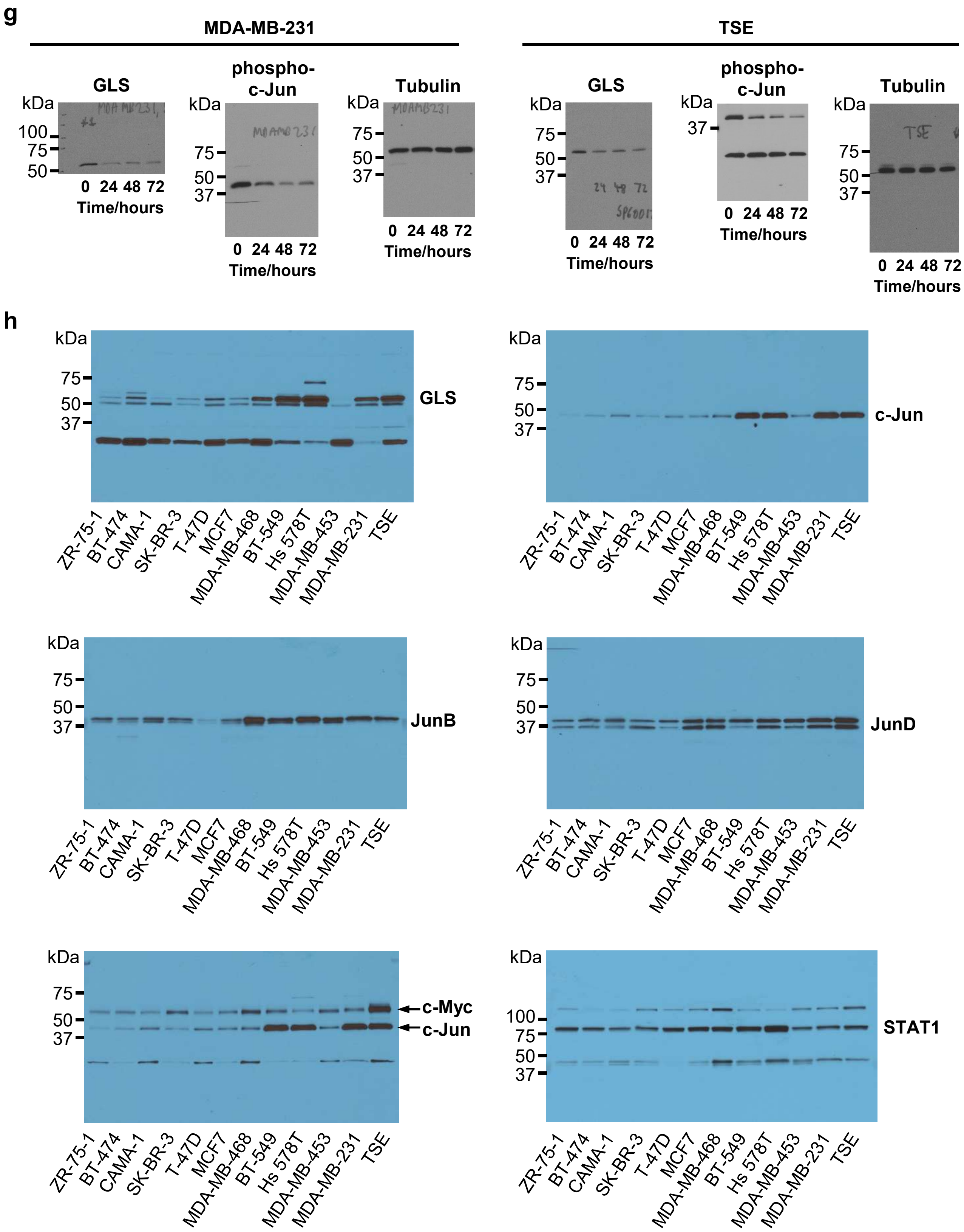

Supplementary Figure 16 (continued). (g) SP600125 (15 μM) treatment timecourse for MDA-MB-231 and TSE cell lines. (h) Breast cancer cell line panel.

h (continued)

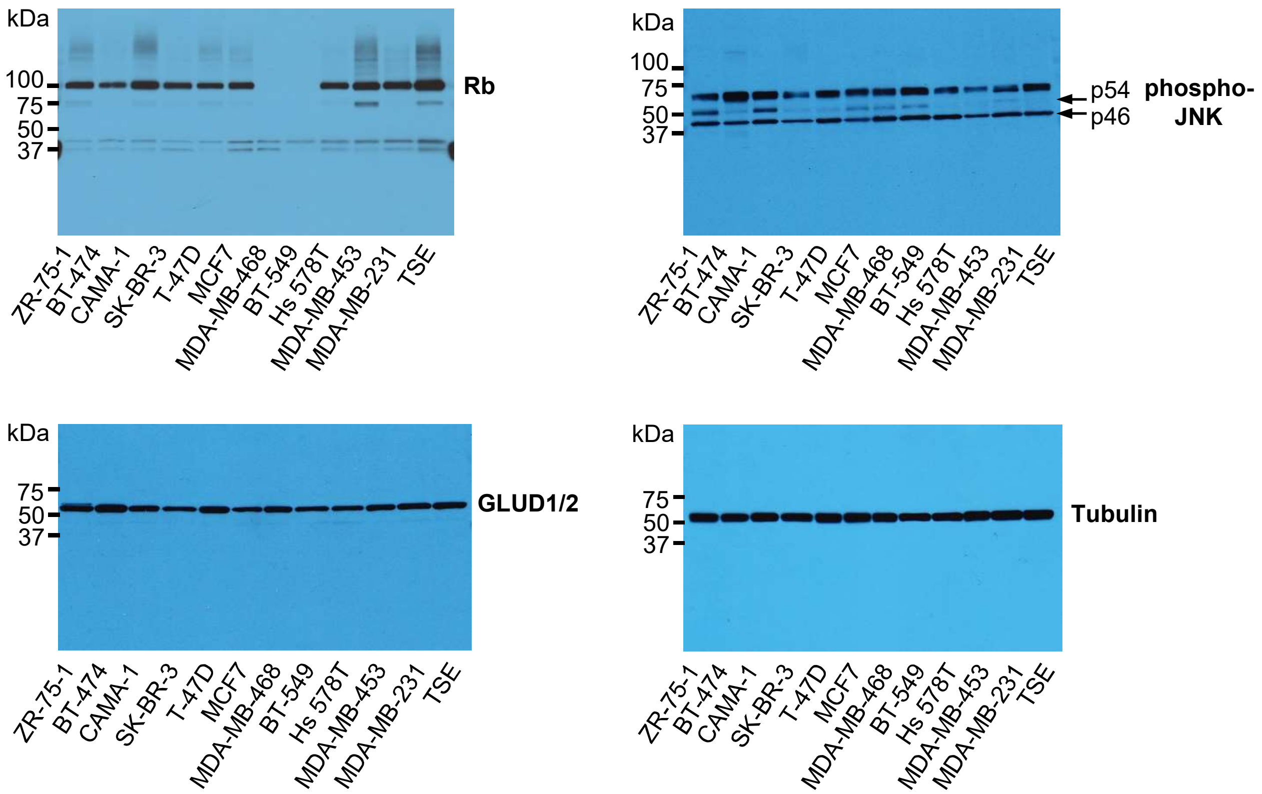
kDa
100
75
50
37
Rb
ZR-75-1
BT-474
CAMA-1
SK-BR-3
T-47D
MCF7
MDA-MB-468
BT-549
Hs 578T
MDA-MB-453
MDA-MB-231
TSE
kDa
100
75
50
37
p54
p46
phospho-
JNK
ZR-75-1
BT-474
CAMA-1
SK-BR-3
T-47D
MCF7
MDA-MB-468
BT-549
Hs 578T
MDA-MB-453
MDA-MB-231
TSE
kDa
75
50
37
GLUD1/2
ZR-75-1
BT-474
CAMA-1
SK-BR-3
T-47D
MCF7
MDA-MB-468
BT-549
Hs 578T
MDA-MB-453
MDA-MB-231
TSE
kDa
75
50
37
Tubulin
ZR-75-1
BT-474
CAMA-1
SK-BR-3
T-47D
MCF7
MDA-MB-468
BT-549
Hs 578T
MDA-MB-453
MDA-MB-231
TSE

IC_{50} values for inhibition of proliferation by BPTES

Cell line	IC_{50}/μM
ZR-75-1	20.7
BT-474	14.7
CAMA-1	>40
SK-BR-3	38.1
T-47D	20.3
MCF7	15.7
MDA-MB-468	18.6
BT-549	18.7
Hs 578T	3.1
MDA-MB-453	14.7
MDA-MB-231	1.8
TSE	7.8

Supplementary Table 1. IC_{50} values for inhibition of proliferation by BPTES for breast cancer cell lines. Proliferation assays were carried out for each cell line under various concentrations of BPTES, and data fitted using SigmaPlot. Representative dose curves are shown in Fig. 5f.

Correlation coefficients for c-Jun up-regulated transcripts and *GLS*

Gene	Spearman	Pearson
EGFR	0.488	0.083
ZEB2	0.426	0.155
FGF7	0.364	0.209
VIM	0.362	0.255
CYP1B1	0.316	0.114
CXCR4	0.314	0.221
MMP1	0.292	0.183
AXL	0.277	0.084
FN1	0.279	0.127
CSF2	0.248	0.107
HBEGF	0.235	0.074
CD44	0.225	0.113
GSTA1	0.220	0.134
BGN	0.157	0.083
GSN	0.150	0.110
SERPINE1	0.149	0.061
CDKN2A	0.137	0.265
COL1A2	0.129	0.008
MGP	-0.018	0.012
TIMP1	-0.082	-0.085

Correlation coefficients for c-Jun down-regulated transcripts and *GLS*

Gene	Spearman	Pearson
STARD10	-0.489	-0.251
NINJ1	-0.427	-0.348
IGFBP2	-0.367	-0.131
KRT19	-0.341	-0.223
RHOD	-0.282	-0.114
ITGA3	-0.171	-0.062
CDKN1B	-0.130	-0.137
CKB	-0.088	-0.017
TRIP6	-0.031	0.040
DDIT3	-0.025	0.020
CDKN1A	-0.020	-0.051

Supplementary Table 2. Correlation coefficients for the *GLS* transcript and established c-Jun target transcripts in invasive breast cancer. Summary of Spearman and Pearson correlation coefficients for the scatter plots shown in Supplementary Figs. 12 and 13. Plots were prepared and correlation coefficients determined using the cBioportal suite of tools, and data from The Cancer Genome Atlas (TCGA) Breast Invasive Carcinoma (TCGA, provisional) dataset.
